# Claudin-4 Modulates Autophagy via SLC1A5/LAT1 as a Mechanism to Regulate Micronuclei

**DOI:** 10.1158/2767-9764.CRC-24-0240

**Published:** 2024-07-02

**Authors:** Fabian R. Villagomez, Julie Lang, Fredrick J. Rosario, Daniel Nunez-Avellaneda, Patricia Webb, Margaret Neville, Elizabeth R. Woodruff, Benjamin G. Bitler

**Affiliations:** 1 Division of Reproductive Sciences, Department of Obstetrics and Gynecology, School of Medicine, University of Colorado, Anschutz Medical Campus, Aurora, Colorado.; 2 Department of Immunology and Microbiology, University of Colorado Anschutz Medical Campus, Aurora, Colorado.; 3 Deputy Directorate of Technological Development, Linkage, and Innovation, National Council of Humanities, Sciences, and Technologies, Mexico City, Mexico.

## Abstract

Genome instability is a hallmark of cancer crucial for tumor heterogeneity and is often a result of defects in cell division and DNA damage repair. Tumors tolerate genomic instability, but the accumulation of genetic aberrations is regulated to avoid catastrophic chromosomal alterations and cell death. In ovarian cancer tumors, claudin-4 is frequently upregulated and closely associated with genome instability and worse patient outcomes. However, its biological association with regulating genomic instability is poorly understood. Here, we used CRISPR interference and a claudin mimic peptide to modulate the claudin-4 expression and its function *in vitro* and *in vivo*. We found that claudin-4 promotes a tolerance mechanism for genomic instability through micronuclei generation in tumor cells. Disruption of claudin-4 increased autophagy and was associated with the engulfment of cytoplasm-localized DNA. Mechanistically, we observed that claudin-4 establishes a biological axis with the amino acid transporters SLC1A5 and LAT1, which regulate autophagy upstream of mTOR. Furthermore, the claudin-4/SLC1A5/LAT1 axis was linked to the transport of amino acids across the plasma membrane as one of the potential cellular processes that significantly decreased survival in ovarian cancer patients. Together, our results show that the upregulation of claudin-4 contributes to increasing the threshold of tolerance for genomic instability in ovarian tumor cells by limiting its accumulation through autophagy.

**Significance:**

Autophagy regulation via claudin-4/SLC1A5/LAT1 has the potential to be a targetable mechanism to interfere with genomic instability in ovarian tumor cells.

## Introduction

Epithelial ovarian cancer (EOC) is a heterogeneous spectrum of diseases that includes high-grade serous (HGSOC), low-grade serous (LGSOC), mucinous (MOC), endometrioid, and clear cell ovarian carcinomas (1), with HGSOC being the most common. Patients with HGSOC have less than 50% 5-year relative overall survival due to the development of therapy resistance. Initially, patients with HGSOC respond to standard care (surgery debulking, carboplatin, paclitaxel) and maintenance treatments (e.g., PARPi, or PARP inhibitors). However, approximately 80% eventually develop therapy resistance. This occurs due to the acquisition of cellular mechanisms that enable tumor cells to counteract the effects of cancer therapy (1–5).

Genomic instability is one of the factors contributing to tumor progression, intratumoral heterogeneity, and the development of resistance to cancer therapy (6–9). It arises from alterations in cell cycle progression and DNA damage repair mechanisms, resulting in an increased rate of mutations and generation of chromosomal modifications, such as amplifications, translocations, and micronuclei formation. Genome instability is a hallmark of cancer, indicating that this characteristic occurs during cancer development, is essential for tumor progression, and is thus tolerated by tumor cells (10–12). For example, tumors comprise heterogeneous cell subpopulations, exhibiting diverse and unique genomic alterations. These tumor cells can restrict the accrual of those alterations to prevent severe chromosomal transformations, indicating the existence of a threshold tolerance for genomic instability within the tumor. Thus, exceeding this critical threshold renders the tumor cells susceptible to cell death. However, the cellular mechanisms regulating such tolerance are not well-known (6, 11–16).

Claudin-4 is aberrantly expressed in most epithelial ovarian carcinomas (17–19), and in up to 75% of HGSOC tumors, where it functions as a driver in the development of resistance to cancer therapeutics, ultimately leading to worse patient outcomes (2). This protein is also closely associated with genomic instability in ovarian cancer (2). For instance, ovarian tumors expressing claudin-4 exhibit stronger resistance to the accumulation of genetic alterations, potentially rendering tumor cells resistant to cell death by preventing excessive accrual of genome instability. This suggests a cellular protective mechanism mediated by claudin-4 to limit the accumulation of genetic aberrations (2). The biological function of this protein is traditionally proposed to be related to tight junctions (TJ). Consequently, a key function of TJs, including claudin-4, is the regulation of permeability (20, 21). However, claudin-4 also participates in other cellular functions, including migration, mitosis, apoptosis, and DNA damage repair (2, 17, 22, 23).

Nevertheless, despite the well-recognized clinical significance of claudin-4 in ovarian cancer (2, 17–19, 23), the molecular mechanisms by which it promotes the development of therapy resistance, as well as its association with genomic instability, remain poorly understood. Here, we modulated claudin-4 in different ovarian cancer cells (HGSOC cells, OVCAR3, which do express claudin-4; LGSOC cells, OVCAR8, which do not express claudin-4; and MOC cells, OVCA429, which do express claudin-4; ref. 1) *in vitro* and *in vivo* [Patient-derived xenograft (PDX)-Humanized Immune System (HIS) mice; patient-derived xenograft-human immune system mice] systems to study the function of claudin-4. We found that claudin-4 forms an axis with the amino acid transporters SLC1A5 and LAT1 to regulate autophagy, facilitating the elimination of a product of genome instability, micronuclei, thus preventing the accumulation of genomic instability in ovarian cancer.

## Material and Methods

### Cell lines, cell culture, mycoplasma testing, and viral vectors

Human-derived cells, OVCA429 (RRID:CVCL_3936), OVCAR3 (RRID:CVCL_0465), OVCAR8 (RRID:CVCL_1629) were obtained for The University of Colorado Gynecologic Tissue and Fluid Bank and cultured in RPMI-1640 medium (Gibco, Thermo Fisher Scientific, Cat. # 11875) plus 10% heat-inactivated fetal bovine serum (Phoenix Scientific, Cat. # PS-100, Lot # 20055-01-01) and 1% penicillin/streptomycin (Corning, Cat. # 30-002-CI) at 37°C and 5% CO_2_. HEK293-FT (RRID:CVCL_6911) were cultured similarly but in DMEM medium (Gibco, Thermo Fisher Scientific, Cat. # 11995040). Cells were only cultured for up to 20 passages and were tested for mycoplasma during the time of experiments (last tested on August 16, 2023) using the Mycoplasma PCR Detection Kit (Sigma-Aldrich, cat. # MP0035).

### Inhibition of claudin-4 expression by CRISPRi

Stable transfectant OVCA429 and OVCAR3-dCas9 cells were generated by lipocomplexes (Lipofectamine 2000, ThermoFisher, cat: 11668-019; vector: pB-CAGGS-dCas9-KRAB-MeCP2, RRID:Addgene_110824) following the manufacturer’s instructions. Selection was carried out by antibiotic resistance (blasticidin). A gRNA (forward: GCT​GGC​TTG​CGC​ATC​AGG​AC; reverse: GTC​CTG​ATG​CGC​AAG​CCA​GC) specific for human *CLDN4* (claudin-4) was generated in the Broad Institute portal (https://portals.broadinstitute.org/gppx/crispick/public). Subsequently, the pCRISPRia-v2_base (TagBFP) vector was digested (BstXI and BlpI) to insert the gRNA by ligation, followed by cloning using *E. coli* (Stbl3, ThermoFisher, cat: C737303), and cell sorting by flow cytometry (MoFlo XDP100; CU Cancer Center Flow Cytometry Shared Resource).

### Lentivirus production and transduction

The 293FT cells were transfected using lipocomplexes (Lipofectamine 2000, ThermoFisher, cat: 11668-019) containing the viral packaging system of second generation (psPAX2, Addgene, Cat. # 12260, RRID:Addgene_12260; pMD2.G Addgene, Cat. # 12259, RRID:Addgene_12259) as well as the lentiviral construct of interest pCDH-EF1a-mCherry-EGFP-LC3B (RRID:Addgene_170446), respectively. Supernatant of the transfected 293 FT cells was collected, filtered (0.45 µm), used, or stored (−80°C).

### Immunoblot

To analyze levels of claudin-4 protein expression, tumor cells were scraped from culture plates in the presence of lysis buffer (30 mmol/L Tris HCl pH 7.4, 150 mmol/L NaCl, 1% Triton X-100, 10% glycerol, 2 mmol/L EDTA, 0.57 mmol/L PMSF, 1× cOmplete Protease Inhibitor Cocktail), placed on a shaker for 10 minutes, and spun at 13,000 rpm for 10 minutes. Protein was separated by SDS-PAGE and transferred to PVDF membrane using the TransBlot Turbo (Bio-Rad). Membranes were blocked with Intercept Blocking Buffer (LI-COR, # 927-60001) for 2 hours at room temperature. Mouse anti-human claudin-4 (Thermo Fisher Scientific Cat. # 32-9400, RRID:AB_2533096, 1:500 dil); rabbit anti-human β-actin (Abcam Cat. # ab6276, RRID:AB_2223210,1:10,000 dil); rabbit anti-LC3 A/C (Cell signaling Cat. # 4108, RRID:AB_2137703, 1: 1,000 dil); rabbit Phospho-p70 S6 Kinase (Cell signaling Cat. # 9205, RRID:AB_330944, 1:1,000 dil); rabbit anti-p70 S6 Kinase (Cell signaling Cat. # 2708, RRID:AB_390722, 1:1,000 dil) primary antibody incubation was performed overnight at 4°C. Membranes were washed three times for 5 minutes each in TBST (50 mmol/L Tris pH 7.5, 150 mmol/L NaCl, 0.1% Tween-20), followed by secondary antibodies for 2 hours at room temperature. Membranes were washed again five times for 5 minutes each in TBST. For fluorescent detection, bands were visualized using the LI-COR Odyssey Imaging System.

### Claudin mimetic peptide synthesis

Claudin mimetic peptide (CMP) structure and synthesis, as previously reported (24).

### Cell cycle by flow cytometry

3 × 10^5^ cells were seeded onto six-well plates. The next day, cells were washed (sterile PBS 1×) and the media was changed. After 24 and 48 hours incubation, cells were detached (trypsin 0.25 mmol/L), centrifuged (1,800 rpm/5 minutes), and washed with PBS followed by centrifugation. PBS was discarded and cells were fixed using cold ethanol 70% (ethanol, milliQ water, v/v) for 30 minutes (4°C) followed by centrifugation (1,200 rpm/5 minutes/4°C). Afterward, cells were washed twice with PBS and the PBS was discarded after centrifugation (1,200 rpm/5 minutes/4°C). Cells were treated with RNAse A (50 µL/100 µmol/L) for 30 minutes at RT and then stained with propidium iodide 300 µL (50 µmol/L). All analyses were carried out in the Cancer Center Flow Cytometry Shared Resource, University of Colorado Anschutz Medical Campus.

### Autophagy flux by flow cytometry

2 × 10^5^ HGSOC cells were seed onto six-well plates (2 mL complete RPMI medium) and treated [CMP, 400 mmol/L; L-g-glutamyl-p-nitroanilide (GPNA), Sigma Cat. # G6133, 25 mmol/L, dissolved in complete RMPI medium; BCH, Tocris Cat. # 5027, 5 mmol/L, dissolved in milli Q water, 37°C] for 24 and 48 hours. Subsequently, cells were detached (trypsin 0.25 mmol/L) and fixed (PBS-PFA 1%).

### Immunofluorescence

Cells were seeded onto 24-well plates containing sterile coverslips (12 mm; 1,5/1/2) and treated with chloroquine (CQ, 40 µmol/L) and rapamycin (8 µmol/L) for 24 hour (1 mL RPMI complete medium). To limit L-glutamine, we used 1 mL complete RPMI 1640 medium, no glutamine (Catalog number: 21870076) for 24 hours. Subsequently, cells were fixed with paraformaldehyde at 4% (PBS 1×) for 10 minutes, followed by permeabilization (30 minutes, 0.1% Triton X-100, PBS 1×). Blocking was carried out by 2-hour incubation with BSA at 5% (PBS 1×, room temperature, RT, shaking). Primary antibodies (mouse, anti-claudin-4, ThermoFisher, cat: 32-9400, RRID:AB_2533096, at 1:200 dilution; rabbit; rabbit anti-Lamin B1, proteintech, Cat. # 66095-1-Ig, RRID:AB_11232208, at 1:800 dil; mouse, anti-Lamin A/C, Cell signaling, Cat. # 4777, RRID:AB_10545756, at 1:100 dil; rabbit, anti-SLC1A5, abcam, Cat. # ab187692, at 1:100 dil; rabbit, anti-LAT1, Cell signaling, Cat. # 5347, RRID:AB_10695104, at 1:100 dil) were incubated (BSA at 2%, PBS 1×) overnight at 4°C with shaking. Secondary antibodies (AlexaFluor546 anti-mouse, ThermoFisher, cat: A-11030, RRID:AB_2737024, at 2 µg/mL; AlexaFluor647 anti-rabbit, ThermoFisher, cat: A32733, RRID:AB_2633282, at 2 µg/mL) were incubated 2 hours/shaking at RT (BSA at 2%, PBS 1×). Nuclei were stained with DAPI at 1 µg/mL (PBS 1×) for 10 minutes. All microscopy acquisition (FV1000, Olympus) was carried out in the Neurotechnology Center, University of Colorado Anschutz Medical Campus.

### Live-cell imaging

2 × 10^5^ cells were seeded onto glass bottom dishes (35 mm, No 1.5; MatTek, Cat. # P35G-1.5-14-C) and 2 mL RMPI complete medium without phenol red (ThermoFisher, Cat. # 11835030). Nuclei were stained using Hoechst 1 µM (ThermoFisher, 62249). All microscopy acquisition (FV1000, Olympus) was carried out in the Neurotechnology Center, University of Colorado Anschutz Medical Campus.

### Metabolomics

Global nontargeted metabolomics was completed as described previously (25). 1 × 10^6^ HGSOC cells were seeded onto six-well plates with complete RPMI media (2 mL) and incubated for 24 hours. Cells were detached using trypsin (0.25 mmol/L) and washed using cold PBS 1×, and centrifuged (1,000 rpm/5 minutes; twice). Likewise, the supernatant was centrifuged (2,000 rpm/5 minutes) or filtered through a membrane of 0.45 µm. Samples were stored until analysis (−80°C). Ultrahigh-performance liquid chromatography-mass spectrometry metabolomics was performed by the University of Colorado School of Medicine Biological Mass Spectrometry Facility (RRID: SCR_021988).

### Amino acid transport

4 × 10^5^ OVCAR3 cells were seeded onto six-well plates [six replicates wild-type (WT), six replicates claudin-4 knockdown (KD) cells] with complete RPMI complete media (2 mL) and incubated for 24 hours. Cells were washed twice with warmed PBS 1×. Briefly, system L-amino acid transporter (LAT) activity was determined as the 2-amino-2-norbornanecarboxylic acid (BCH)-inhibitable uptake of [3H] leucine, as previously reported (26). Cells were washed twice with 3 mL of Tyrode solution at 37°C with or without Na^+^ and BCH (64 μmol L^−1^) and then incubated for 1.5 minutes in 1.0 mL of Tyrode solution (with or without Na^+^ and BCH) containing [3H] leucine in final concentrations of 0.0125 µmol/L. Each condition was studied in triplicate. Uptake was terminated by washing three times with 2 mL of ice-cold Tyrode solution without Na^+^ and BCH. Cells were lysed in distilled H_2_O for 1 hour and then denatured in RIPA buffer. The water containing the tracers released from the cells was mixed with scintillation fluid and counted in a β-counter.

### Generation of HIS-BRGS mice and chimerism evaluation

#### Stem cell isolation

Hematopoietic stem cells were isolated from peripheral blood mononuclear cells prepared from clinically rejected CB units from the University of Colorado Cord Blood bank (Clinimmune Labs) using CD34^+^ magnetic Miltenyi beads, and expanded in short-term cultures with IL6 (10 ng/mL), SCF (40 ng/mL), and FLt3L (20 ng/mL). CD34^+^ cells, harvested between days 4 and 6, were frozen in 90% FCS/10%DMSO and stored at −80°C prior to injection into neonate pups. Investigators were blinded by donor identities, and the donors provided written informed consent. The studies were performed in accordance with ethical guidelines detailed by the Declaration of Helsinki and in compliance with the University of Colorado Institutional Review Board (COMIRB # 16-0541).

#### HIS-BRGS mice and chimerism evaluation

To generate human immune system mice, neonatal (d1-3) BRGS (BALB/c*Rag2*^nul*l*^*IL2Rg*^null^*Sirpa*^NOD^) pups, obtained from the laboratory of James Di Santo, were irradiated with 300 rads 2 to 6 hours prior to injection with 0.2 to 0.6 × 10^6^ expanded then thawed CD34^+^ cells. The number of cells injected is equivalent to 50,000 fresh CD34^+^ cells per mouse, i.e., cell count prior to *in vitro* expansion. Mice were bred and engrafted in the University of Colorado Denver Anschutz Medical Campus vivarium with prior Institutional Animal Care and Use Committee (IACUC) protocol and in a facility accredited by the American Association for Accreditation of Laboratory Animal Care. BRGS mice, both breeders and engrafted, were maintained on an alternating biweekly Septra-enriched (Uniprim) diet. Mice were injected in the facial vein, liver, or both. Humanized mice were generated in the Pre-clinical Human Immune System Mouse model Shared Resource, University of Colorado Anschutz Medical Campus.

All mouse work was performed in accordance with the Guide for the Care and Use of Laboratory Animals and was approved of by the University of Colorado’s Institutional Animal Care and Use Committee (IACUC protocol # 283). HIS mice were i.p. injected with 5 × 10^6^ cells of a developed PDX model, PDX GTFB 1016, described previously (27). Briefly, this model is derived from a primary tumor collected from a patient diagnosed with stage IIIC, who was described to have a high volume of ascites, peritoneal carcinomatosis, and was chemonaïve at the time of sample collection. TP53 and BRCA2 mutations were identified, and we subsequently transfected the cells with a GFP-luciferase tag, enabling tumor tracking by *In Vivo* Imaging (IVIS, Perkin Elmer), as described previously (28, 29). Tumors were allowed to establish for 3 weeks prior to treatment initiation. After this time, mice were treated with CMP (4 mg/kg, i.p.) every 2 days. Control mice were treated with respective vehicles on the same treatment schedule PBS, i.p. (CMP vehicle). Treatment occurred for 28 days, and mice were IVIS scanned weekly to assess tumor development. One day after the last treatment, mice were euthanized via CO_2_ inhalation and cervical dislocation, and tissues were collected.

Blood and spleen were stained and analyzed via flow cytometry, as described previously (30). Blood samples for chimerism assessment were collected on a Bio-Rad Yeti 5-laser flow cytometer, and spleen and tumor harvest samples were collected on the Cytek Aurora 5-laser spectral cytometer at the University of Colorado Cancer Center Flow Cytometry Shared Resource. All data were analyzed using FlowJo software.

### Statistical considerations

ImageJ (NIH) and Prism software (v9.0) were used for microscopy and statistical data analysis, respectively. Three biologically independent experiments were conducted. Unpaired *t* and Mann–Whitney tests, Kruskal–Wallis test, and one-way ANOVA with Dunn’s or Tukey’s multiple comparison test based on normal data distribution and number of variables were used. The level of significance was *P* < 0.05.

### Ethical approval

Stem Cell Isolation and patient-derived tumors for PDX was performed in compliance with the University of Colorado Institutional Review Boards, approved COMIRB protocol # 16-0541 (Stem Cell Isolation) and protocol [# 07-0935, (27)]. All mouse work was performed in accordance with the Guide for the Care and Use of Laboratory Animals and the protocol was approved by the University of Colorado’s Institutional Animal Care and Use Committee (IACUC protocol # 283).

### Data availability

Data generated in this study are included in this manuscript and in its Supplementary Material. Data mining and flow cytometry raw data are available upon request to the corresponding author.

## Results

### Micronuclei are a common form of genomic instability that are impacted by claudin-4 disruption in ovarian tumor cells

**Figure 1:**
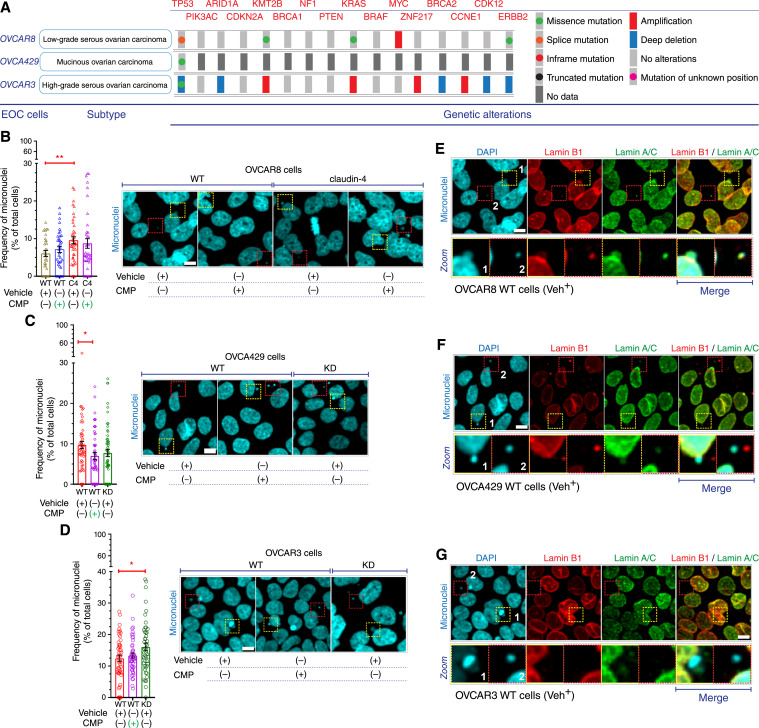
Characterization of micronuclei during claudin-4 manipulation in HGSOC cells. HGSOC cells were treated with CMP (400 µmol/L; 48 hours). Then, cells were fixed and stained with DAPI to mark DNA and the components of the nuclear lamina, lamin B1 and lamin A/C. Subsequently, a morphometric characterization was performed through confocal microscopy. **A,** Illustration indicating the histological subtype of EOC cells used as *in vitro* system, as well as certain genetic alterations. **B,** (Left), frequency of micronuclei during claudin-4 overexpression; (right), confocal images (maximum projections) highlighting (dotted squares) micronuclei. Similar data is presented for claudin-4 downregulation in OVCA429 (**C**) and OVCAR3 cells (**D**). (*n* = 4,134 OVCAR8 cells; *n* = 3,315 OVCA429 cells; *n* = 4,653 OVCAR3 cells; Two-tailed Mann–Whitney test). **E,** Selected confocal images (OVCAR8 WT cells treated with the vehicle from (**B**) showing lamin B1 and lamin A/C. It is highlighted the lack of nuclear lamina in some micronuclei. Similar data is presented in (**F** and **G**) for OVCA429 and OVCAR3 cells, respectively. (three independent experiments; Kruskal–Wallis test with Dunn's multiple comparisons, *P* < 0.05). Graphs show mean and SEM, scale bar, 10 µm.

To investigate the functional association of claudin-4 with genomic instability in ovarian cancer and to better represent the known heterogeneity of these diseases (1), we modulated claudin-4 expression in three diverse EOC cells: OVCAR3 and OVCA429 (as claudin-4 downregulation system) and OVCAR8 (as claudin-4 overexpression system) using CRISPR interference and lentiviral transduction, respectively (see Supplementary Fig. S1A). Additionally, we employed a CMP with documented antiovarian tumor activity (17), known to mimic a conserved sequence in claudin-4, resulting in its mislocalization (see Supplementary Fig. S1B; ref. 24). The heterogeneity of the selected EOC cells can be observed through their histological classification: HGSOC (OVCAR3 cells), LGSOC (OVCAR8 cells), and MUC (OVCA429 cells), each of which also exhibits distinct genetic alterations (Fig. 1A; ref. 1).

Micronuclei, which are DNA-containing structures separated from the primary nucleus and encapsulated by the nuclear envelope, serve as indicators of genomic instability and are prone to formation in ovarian tumor cells following genotoxic insults (31, 32). We cultured claudin-4-modulated cells and stained the EOC cells to mark DNA (DAPI) and the main components of the nuclear envelope (lamin A/C and lamin B1; ref. 33). Subsequently, we examined micronuclei as indicators of genomic instability through morphometric characterization. Micronuclei were identified in all ovarian tumor cells tested, with more observed in OVCAR3 WT cells, followed by OVCA429 WT and then OVCAR8 WT cells (Fig. 1B–D). This confirms that this type of chromosomal alteration is a common characteristic of ovarian tumor cells (32). Additionally, it is suggested that an association between the loss of p53 in OVCAR3 cells (Fig. 1A) along with their increased frequency of micronuclei (Fig. 1B; compared to OVCA429 and OVCAR8) and the reported susceptibility of nuclear envelope rupture due to *TP53* loss in tumor cells (34). Specifically, regarding the effect of claudin-4 modulation on the same genetic alteration, we observed significant modifications in the frequency of these micronuclei in EOC cells. Claudin-4 overexpression in OVCAR8 (C4) led to a meaningful increase in the number of micronuclei (Fig. 1B), similar to the numbers observed in OVCA429 WT (Fig. 1C) but fewer than in OVCAR3 WT cells (Fig. 1D). Furthermore, downregulation of claudin-4 KD correlated with a significant increase of micronuclei only in OVCAR3 cells (Fig. 1D), while downregulation of claudin-4 showed a trend of fewer micronuclei in OVCA429 (Fig. 1C). Additionally, the effect of CMP treatment was evident only in OVCA429 cells, with reduced levels of micronuclei (Fig. 1C). These initial results reflect the heterogeneity of the tested cells (Fig. 1A) and highlight an association between the types of *TP53* mutations, potentially impacting micronuclei formation (34). For example, OVCAR3 cells display missense mutation and deep deletion, resulting in the highest frequency of micronuclei, while OVCA429 and OVCAR8 harbor missense mutation and splice mutation, respectively (Fig. 1A; ref. 1). Micronuclei can originate from different phases of the cell cycle, such as interphase and mitosis (35–37), and it is reported that claudin-4 participates in the cell cycle (23), a cellular process where p53 acts as a master regulator (38). Particularly, the downregulation of claudin-4 in OVCAR3 cells, synchronized under starvation conditions, resulted in cells arrested in the G2/M phase (23), which correlated with more micronuclei quantified in OVCAR3 claudin-4 KD cells (Fig. 1D). On the other hand, the overexpression of claudin-4 has been reported to increase proliferation in breast cancer cells (22). Thus, since modulation of claudin-4 expression (overexpression and downregulation) resulted in significant changes in the incidence of micronuclei, it is feasible that the levels of this protein could play a major role in their formation, potentially throughout different phases of the cell cycle. Furthermore, the significant decrease in micronuclei observed in OVCA429 WT cells treated with CMP but not in the other tested cells, suggests a potential unique effect of CMP in modifying the claudin-4-interacting proteins in these cells compared to other (39). This is feasible given that CMP can induce the mislocalization of claudin-4 through its effect on the mimicry region in claudin-4 (see Supplementary Fig. S1B; ref. 24), which, in turn, harbors a predicted phosphorylation site—an essential protein modification for protein–protein interactions (40).

Interestingly, our morphometric analysis also revealed that some micronuclei lacked components of the nuclear envelope (lamin A/C and lamin B1; Fig. 1E–G), a phenomenon reported to lead to the collapse of micronuclei and the subsequent release of DNA into the cytoplasm (37, 41–43). This potential collapse of micronuclei prompted us to explore its consequences, particularly the release of DNA into the cytoplasm, which can trigger a type I interferon response mediated by cGAS-STING (37, 41–43). However, it is reported that this signaling pathway is inhibited in ovarian tumor cells (44). Consequently, we hypothesized that the association claudin-4 with micronuclei could also be related to their elimination, thereby preventing DNA release from the collapsing micronuclei and its detection by cGAS. For example, it is known that the primordial function of cGAS-STING signaling is the activation of autophagy to clear cytosolic DNA (45), and it has been reported that autophagy can remove micronuclei in osteosarcoma cells (46).

### Claudin-4 participates in autophagy, which facilitates the engulfment of micronuclei in ovarian tumor cells

To get more insights into our hypothesis, we performed immunoblotting for the autophagy marker, LC3 A/B, which can be found in two forms: LC3 A/B-i (cytosolic) and LC3 A/B-ii (membrane bound). The lipidated LC3 A/B-ii form binds to autophagosomes, which are subsequently degraded as autophagosomes and move intracellularly and fuse with lysosomes. Our immunoblotting analysis indicated that disruption of claudin-4 altered the levels of LC3 A/B, especially during claudin-4 downregulation (see Supplementary Fig. S1C). Additionally, we treated OVCA429 WT cells with rapamycin (an upstream activator of autophagy through inhibition of mTORC1) and chloroquine (a downstream inhibitor of autophagy through blocking vesicular trafficking) to shed light on the levels of LC3 A/B-i and LC3 A/B-ii and the potential activation status of autophagy in EOC cells. We observed a decrease in LC3 A/B-ii levels during rapamycin-induced autophagy, while chloroquine-inhibited autophagy resulted in an increase in LC3 A/B-ii levels (see Supplementary Fig. S1D). Although our initial experiments did not conclusively indicate whether autophagy was activated or inhibited during claudin-4 manipulation, they suggest that disruption of claudin-4 could indeed alter autophagy activity.

In support of such an assertion, we determined the phosphorylation status of p70-S6 kinase, a downstream target of mTORC1 (a negative regulator of autophagy). Our findings showed an increase in p70-S6 with claudin-4 overexpression and a decrease during claudin-4 downregulation (see Supplementary Fig. S1E–S1G). This suggests that mTORC1 activity could be affected by claudin-4 disruption as well. Specifically, reduced levels of p70-S6 kinase phosphorylation during claudin-4 KD (see Supplementary Fig. S1F and S1G) suggest potential inhibition of mTORC1, thereby impacting autophagy. Together, these results imply that claudin-4 may participate in the regulation of autophagy either through upstream or downstream effects in different EOC cells.

To measure autophagy more directly, we generated EOC cells expressing the tandem GFP-mCherry-LC3 to measure the activity of this cellular process (autophagy flux) using flow cytometry and confocal microscopy, as previously reported (47). Briefly, GFP-mCherry-LC3 is observed to localize to autophagosomes, which then move intracellularly and merge with lysosomes. This fusion event results in a decrease in the pH within the vacuolar lumen, leading to the subsequent loss of GFP fluorescence due to quenching. In contrast, mCherry fluorescence remains unaffected by changes in the pH. Thus, an increase in the number of mCherry-positive cells originating from double-positive GFP-mCherry cells serves as an indicator of autophagy flux or activity (see Supplementary Fig. S2A). To validate our strategy, EOC-GFP-mCherry-LC3 cells were treated with CQ and rapamycin to block and activate autophagy, respectively. These cells responded appropriately to these stimuli, showing inhibition of autophagy flux during CQ treatment and the opposite effect during rapamycin treatment (see Supplementary Fig. S2B). Subsequently, we directly evaluated the effect of claudin-4 manipulation and the impact of CMP treatment on the autophagy flux.

**Figure 2:**
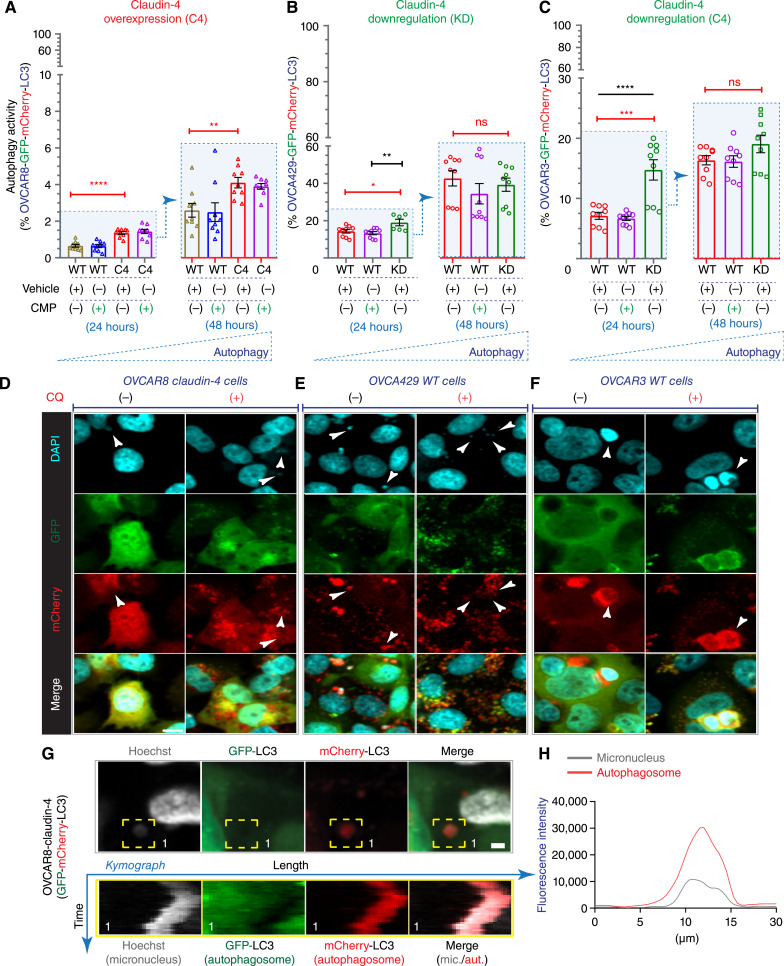
Claudin-4 links genomic instability to autophagy in HGSOC cells. We analyzed the functional role of claudin-4, establishing a link between autophagy and micronuclei (indicator of genomic instability) in HGSOC cells. This analysis was conducted both *in vitro* (HGSOC-GFP-mCherry-LC3 expressing cells) and *in vivo* (PDX in a humanized mice model). The assessment involved techniques such as flow cytometry, confocal microscopy (CM), and multispectral immunofluorescence. **A–C,** show percentage autophagy flux in HGSOC cells (flow cytometry) during CMP treatment (400 µmol/L), and claudin-4 genetic manipulation at 24 and 48 hours of culture (four independent experiments; Dotted blue triangles suggest an increase in autophagy). **D–F,** show confocal images of HGSOC cells expressing GFP-mCherry-LC3 and stained with DAPI (white arrow indicates the convergence of autophagy flux (mCherry) with micronuclei. G**,** Top, shows confocal images (from live-cell imaging) highlighting (yellow dotted square) a micronucleus; (bottom), kymographs generated from live-cell confocal imaging (from yellow dotted square). It shows the mobility pattern over time of the same region in every channel (Hoechst, GFP, and mCherry). **H,** Shows a line scan that indicates the fluorescence pattern for the micronucleus and autophagosomes (mCherry) from (**G**). (significance, *P* < 0.05). Graphs show mean and SEM, scale bar, 10 and 5 µm.

Among the EOC cells analyzed, OVCA429 WT (MOC subtype) showed the highest baseline percentage of cells with autophagy flux, then OVCAR3 WT (HGSOC subtype), and to a lesser extent OVCAR8 WT (LGSOC subtype; Fig. 2A–C). The observed autophagy flux in the EOC cells also correlated with TP53 in autophagy (48), and with the specific *TP53* mutations detected among different ovarian tumor cells (Fig. 1A), highlighting the potential impact of different *TP53* mutations on autophagy regulation (49). Importantly, in modulating claudin-4 in EOC cells, we validated its involvement in autophagy. We found that variations in claudin-4 protein levels had a greater impact on its association with autophagy flux compared to any mislocalization effect caused by CMP. The overexpression of claudin-4 in OVCAR8 cells led to a sustained increase in autophagy flux (Fig. 2A; 24 and 48 hours). Downregulation of claudin-4 in OVCA429 and OVCAR3 cells also resulted in increased autophagy flux; however, this increase was not sustained, suggesting that at later stages of cell culture, an additional factor other than claudin-4 downregulation (Fig. 2B and C) may be driving the activation of autophagy, potentially reduced nutrient availability in the chronic setting. This notion is supported by the understanding that nutrient availability decreases in cell culture over time (50), the catabolic nature of autophagy and its induction during conditions of starvation as well as the indirect inhibition of a sensor of nutrient availability, mTORC (as indicated by reduced phosphorylation of p70 S6 kinase; ref. 51) observed during claudin-4 downregulation (see Supplementary Fig. S1F and S1G). Moreover, the sustained increase in autophagy activity observed during claudin-4 overexpression suggests that claudin-4 may influence a key positive or negative element in autophagy signaling. In light of this, the observation that both claudin-4 overexpression and downregulation resulted in increased autophagy in the acute setting (but not in the chronic setting) suggests that claudin-4’s positive effect on autophagy may involve modulation of a key negative regulator of autophagy activation. Therefore, both claudin-4 overexpression and downregulation could induce autophagy.

**Figure 3:**
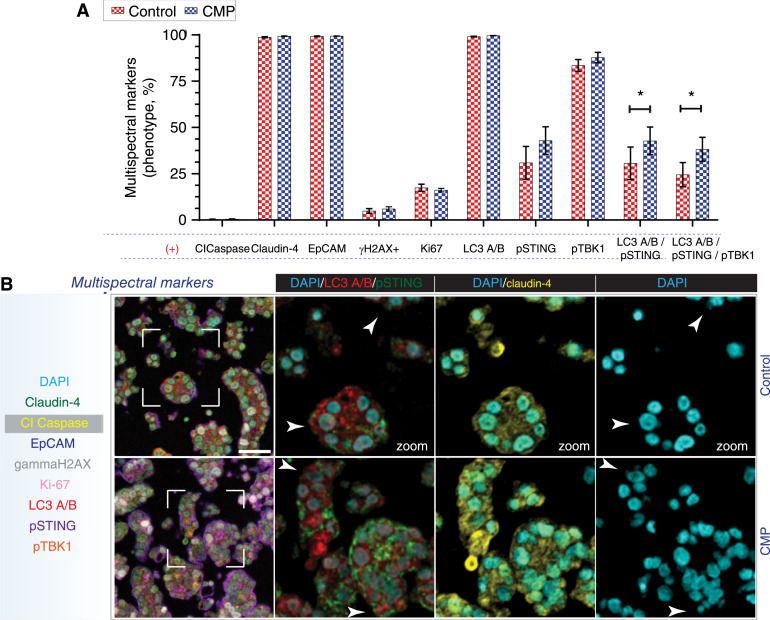
*In vivo* association between indicators of autophagy and genomic instability during CMP treatment. Ascites was obtained from tumor-bearing mice treated or not with CMP (4 mg/kg) and prepared in histogel, followed paraffin embedding and multispectral staining (eight proteins plus DAPI). Afterward, a phenotypic characterization was performed. **A,** Frequency of cells obtained from ascites samples at the end of the study (30 days) showing individual markers as well as cells positive for LC3 A/B and pSTING, and LC3 A/B, pSTING, and pTBK1. **B,** Representative multispectral IF images (all markers used are indicated in the blue-light box) are shown, where a region of interest (white square) is amplified to show specific markers (white arrow are highlighting the overlay of nuclei and the marker of autophagy, LC3 A/B). (Multiple *t* test; significance; *P* < 0.05). Graphs show mean and SEM, scale bar, 50 µm.

Furthermore, we fixed the EOC cells (expressing GFP-mCherry-LC3) to mark the DNA (DAPI) and searched for micronuclei and autophagosomes using confocal microscopy. We discovered that micronuclei are linked with autophagy in all EOC cells, as demonstrated by the spatial alignment of micronuclei with autophagosomes (which are marked with mCherry). Moreover, this correlation was more evident when autophagy flux was inhibited by chloroquine (Fig. 2D–F), strongly suggesting that micronuclei are engulfed by autophagosomes and subsequently degraded through the autophagy flux, given that degradation is a fundamental aspect of autophagy (46, 52). This assumption is strengthened by our observations using confocal live-cell imaging and OVCAR8-claudin-4 cells expressing GFP-mCherry-LC3. We observed that a micronucleus exhibited a movement pattern similar to that of an autophagosome (Fig. 2G; Supplementary Movies S1–S4), with the autophagosome appearing to enclose the micronucleus (Fig. 2H). Together, our findings demonstrate that claudin-4 participates in the autophagy pathway and firmly indicates an association between autophagy and claudin-4’s involvement in genome instability, particularly micronuclei. Additionally, to explore the association of claudin-4 with autophagy *in vivo*, we implanted a reported patient-derived ovarian tumor xenograft (PDX; ref. 27) into a previously established humanized mouse model (PDX-HIS mice) system (30), as described in previous studies. These mice were treated for 30 days with CMP (4 mg/kg; intraperitoneal injection, every 2 days), consistent with previous reported (17). At the conclusion of the study, ascites containing tumor cells and fluid were collected and subjected to paraffin embedding. Following this, multispectral immunofluorescence was employed to characterize the biological effects of targeting claudin-4 with CMP, with a specific focus on indicators related to the autophagy pathway and micronuclei. We observed an increase in the number of tumor cells displaying a phenotypic association of pSTING and LC3 A/B, as well as pSTING, LC3 A/B, and pTBK1. Given the known role of STING in autophagy activation via LC3 (45), these observed phenotypic increases suggest that autophagy was occurring during claudin-4 targeting with CMP (Fig. 3A). Interestingly, we also found a close association between the autophagy marker LC3 A/B and micronuclei (Fig. 3B), which collectively supports the potential involvement of autophagy in clearing micronuclei through a claudin-4-dependent pathway *in vivo*.

### The regulation of amino acid transport correlates with the claudin-4’s association with autophagy and its clinical significance in ovarian cancer

**Figure 4:**
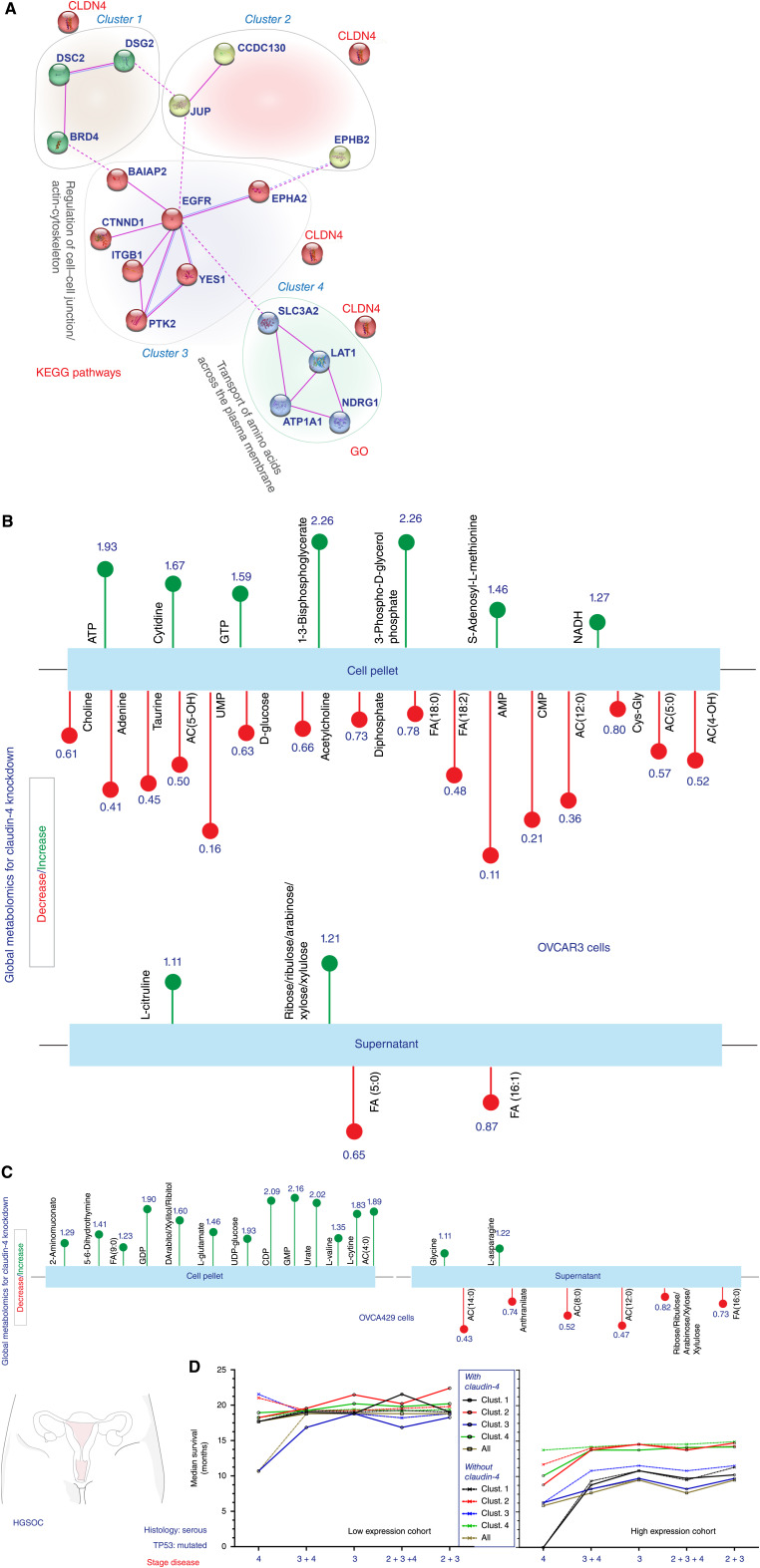
Correlation of amino acids transport with the clinical significance of claudin-4 in ovarian cancer. Reported proteins interacting with claudin-4 in HGSOC cells were employed to construct PPN, aiming to identify key elements and cellular functions associated with the clinical significance of claudin-4 in HGSOC tumors. This analysis utilized publicly available data from HGSOC tumors, sourced from cBioPortal and Kaplan–Meier plotter. **A,** PPN (based on BioID) of claudin-4-interacting proteins (experimentally determined using STRING) and highly mutated proteins (cBioPortal) in HGSOC tumors shows clusters of proteins and distinctive associated cellular functions. **B,** Significantly different metabolites in OVCAR3 cells associated to claudin-4 downregulation (from global metabolomics using cell pellets and supernatants; claudin-4 KD/WT, where values close to 1 are similar results between KD and WT). It also indicates the corresponding mean fold change in the (top) of circles, (red and green; red indicates decreased while greed indicates increase of metabolites). **C,** Same as (**B**) but in OVCA429 cells. **D,** The median survival of HGSOC patients (criteria: p53 mutated; serous histology) was correlated with each identified cluster (based on claudin-4; continue lines) and without claudin-4 (dotted lines; significance, *P* < 0.05; *P* values and false discovery rate is indicated in Supplementary Tables S3 and S4).

Our results demonstrate an association between micronuclei and autophagy mediated by claudin-4. To delve into the molecular mechanisms underpinning this association, we utilized data mining to enrich and identify proteins interacting with claudin-4 in EOC cells via a protein–protein network (PPN) analysis using the STRING server. This search aimed to unveil crucial cellular processes mediated by claudin-4 potentially relevant in ovarian tumors. The basis for this PPN was a set of claudin-4-interacting proteins identified through BioID and previously reported in OVCAR3 cells (39). The PPN was further enriched by incorporating elements with experimentally reported interactions corresponding to each claudin-4-interacting protein identified through BioID (39). Subsequently, the corresponding genes of the total enriched proteins were analyzed in cBioportal (datasets of ovarian tumors) to identify highly mutated elements directly associated with the claudin-4-interacting proteins, revealing BRD4, CCDC130, PTK2, and NDRG1 as the most mutated elements. Notably, our BioID analysis also identified NDRG1 as a potential claudin-4-interating protein in ovarian tumor cells, with more than twice the number of peptides identified for this protein compared to control cells (see Supplementary Table S1). Subsequently, we generated the PPN using those claudin-4-interacting proteins that showed experimental interaction and included the highly mutated elements we identified in The Cancer Genome Atlas ovarian cancer tumors. Afterward, the proteins were clustered based on functionality and cellular processes using gene ontology and KEGG pathways. The hallmark cellular functions were regulation of “cell–cell junctions” and “actin-cytoskeleton,” and “transport of amino acids” (Fig. 4A). This outcome is expected given that claudin-4 is described as a TJ protein (39) and LAT1 (also known as *SLC7A5*) and SLC3A2 transport amino acids (53, 54).

To experimentally support our data mining results, we performed global metabolomics analyses in OVCA429 and OVCAR3 cells (WT vs. claudin-4 KD cells), identifying metabolites significantly altered due to claudin-4 downregulation in EOC cells (see Supplementary Table S2). For example, the most prominently increased metabolites were ATP and GMP in OVCAR3 and OVCA429 cells, respectively (Fig. 4B and C). Notably, functional enrichment analysis using the metabolites significantly altered by claudin-4 downregulation revealed their association with SLC-mediated transmembrane transport and the cell cycle (see Supplementary Fig. S3A), and SLC-mediated transmembrane transport and amino acid transport across the plasma membrane (see Supplementary Fig. S3B). Given the crucial role of amino acids in regulating autophagy activity (54, 55), the transport of amino acids in tumor cells could represent a pivotal factor in claudin-4’s involvement in autophagy-mediated clearance of micronuclei, thereby potentially influencing the clinical significance of claudin-4 in ovarian cancer. This concept is supported by our findings, which show a correlation between the expression of clustered proteins associated with claudin-4 and aggressiveness in ovarian tumors, ultimately resulting in reduced patient survival (Fig. 4D). Furthermore, we utilized these clusters to explore their correlation with aggressiveness in other types of cancer, revealing that all clusters are linked with poorer patient outcomes in breast and lung cancer, though not in stomach cancer. Notably, cluster four exhibited a correlation with decreased relapse-free survival and overall survival in breast, lung, and stomach cancer (Supplementary Fig. S4A–S4C). Thus, our results suggest a link between the association of claudin-4 with genomic instability and autophagy, with the transport of amino acids potentially acting as a regulator of autophagy and thereby impacting the clinical significance of claudin-4 in ovarian cancer.

### Claudin-4 modulates the intracellular distribution of amino acid transporters that regulate autophagy in ovarian tumor cells

Three claudin-4-interacting proteins we reported previously are the amino acid transporters SLC1A5, LAT1, and SLC3A2 (39). The BioID method suggests that claudin-4 and these proteins are in the same protein complex and possibly have a functional relationship (56). It is known that LAT1 forms a heterodimer with SLC3A2 to enable a bidirectional transport system of amino acids along with SLC1A5. This system regulates mTOR upstream in cellular processes such as cell growth and autophagy by controlling the influx and efflux of amino acids (54). In this regulatory process, L-glutamine plays a key role facilitated by its intracellular internalization driven by SLC1A5. Subsequently, L-glutamine serves as a substrate for LAT1, which internalizes essential amino acids while L-glutamine exits the cell. Consequently, this system constitutes a bidirectional transport mechanism for amino acids (54).

**Figure 5:**
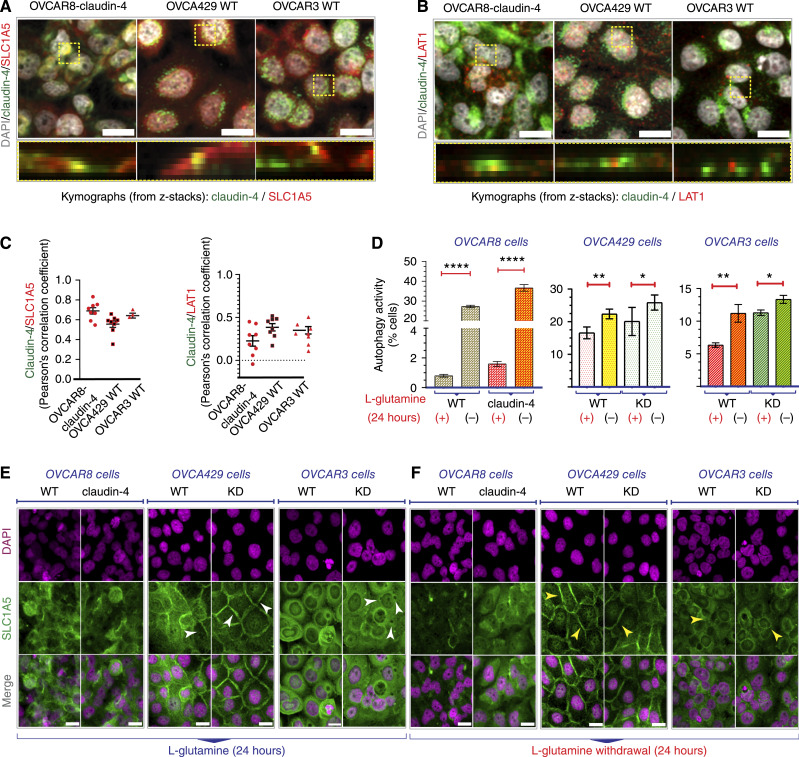
Effect of claudin-4 manipulation on the intracellular distribution of transporters of amino acids. We studied the association of claudin-4 with the amino acid transporters, SLC1A5 and LAT1 (which regulate autophagy) in HGSOC cells using confocal microscopy, immunoblotting, and flow cytometry. **A,** Top, confocal images showing the intracellular distribution of SLC1A5 and claudin-4 in HGSOC cells; bottom, kymographs (from z-stacks and dotted yellow squares) highlighting colocalization. A similar phenotype is shown for LAT1 and claudin-4 in (**B**). The Pearson’s correlation coefficient for SLC1A5 and claudin-4, and LAT1 and claudin-4 is shown in (**C**). **D,** Percentage of HGSOC cells with autophagic flux during L-glutamine withdrawal measured by flow cytometry (two-tailed unpaired *t* test and Mann–Whitney test; three independent experiments; significance, *P* < 0.05). **E** and **F,** show representative confocal images (maximum projections) of the intracellular distribution of SLC1A5 before claudin-4 overexpression (OVCAR8 claudin-4) and downregulation (OVCA429 claudin-4 KD and OVCAR3 claudin-4 KD) in HGSOC cells with and without L-glutamine, respectively. Graphs show mean and SEM, scale bar, 20 µm.

As a result, we employed various approaches to evaluate the impact of claudin-4 on the amino acid transport system and its relationship with autophagy. Initially, we stained EOC cells to visualize SLC1A5, LAT1, and claudin-4 by confocal microscopy. We determined that claudin-4 co-localized with both transporters of amino acids, especially SLC1A5 (Fig. 5A–C), which is consistent with the BioID data (39). We then restricted the availability of L-glutamine in cultured cells and assessed autophagy flux using flow cytometry. All tested EOC cells (OVCAR8, OVCA429, and OVCAR3) exhibited a significant increase in autophagy flux due to L-glutamine limitation (Fig. 5D). This underscores the critical role of this amino acid as a negative regulator of autophagy, as previously documented (54), regardless of EOC subtype and specific genetic alterations (Fig. 1A; ref. 1).

Remarkably, we detected alterations in the intracellular localization of SLC1A5, the amino acid transporter responsible for L-glutamine internalization (54), directly linked to claudin-4 downregulation (in OVCA429 and OVCAR3 cells; Fig. 5E). The most pronounced effect was observed in OVCA429 claudin-4 KD cells, where SLC1A5 was notably concentrated at cell–cell junctions. In contrast, in OVCAR3 claudin-4 KD cells, the same amino acid transporter was excluded from cytoplasmic regions (Fig. 5E), possibly from vacuoles (57). Conversely, the overexpression of claudin-4 in OVCAR8 cells did not result in a noticeable modification of SLC1A5 intracellular distribution. This observation may be attributed to the fact that OVCAR8 cells carry *Myc* gene amplification, unlike OVCAR3 and OVCA429 cells (Fig. 1A; ref. 1), which is known to transcriptionally regulate SLC1A5 and enhance glutamine uptake by inducing SLC1A5 expression (58, 59). Consequently, these results indicate that claudin-4 plays a crucial role in the intracellular localization of SLC1A5 in EOC cells, and reducing the expression of claudin-4 affects the localization of the amino acid transporter, potentially impacting its function. Additionally, we limited the availability of L-glutamine in cell culture and observed the intracellular location of SLC1A5. Limiting L-glutamine in EOC cells also resulted in evident changes in the intracellular distribution of SLC1A5. Particularly, this limitation in OVCA429 WT cells resulted in a phenotype (Fig. 5F, middle) that resembled the effect observed in OVCA429 claudin-4 KD cells regarding the localization of SLC1A5 (Fig. 5E, middle). In OVCAR3 cells, we observed an increased accumulation of SLC1A5 in puncta in the cytoplasm (Fig, 5F, right), possibly vacuoles as previously reported (57). In contrast, in OVCAR8 cells, the overexpression of claudin-4 seemed to prevent changes in SLC1A5 associated with L-glutamine limitation (Fig. 5F, left). These findings suggest a close relationship between claudin-4 expression and amino acid transporters (SLC1A5/LAT1), particularly SLC1A5. Additionally, they support the reported bidirectional transport system involving SLC1A5 and LAT1, which could potentially regulate autophagy (54) in EOC cells.

**Figure 6:**
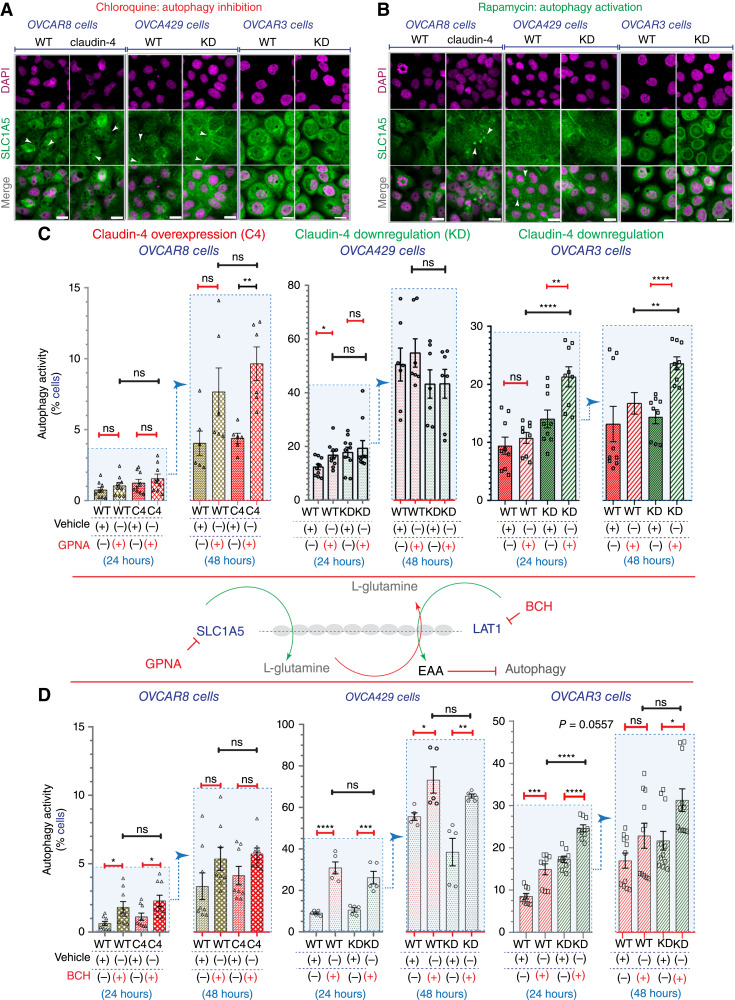
Association of autophagy and the activity of transporters of amino acids. The association of autophagy with the activity of SLC1A5 and LAT1 was evaluated through confocal microscopy and flow cytometry and by using both an activator of autophagy (rapamycin 8 µmol/L, 24 hours) and an inhibitor (chloroquine, 40 µmol/L, 24 hours) as well as the specific inhibition of SLC1A5 and LAT1 using GPNA (25 mmol/L) and BCH (5 mmol/L), respectively. **A,** Confocal images showing SLC1A5 intracellular distribution in HGSOC cells during autophagy blocking autophagy induction (**B**). **C,** Graphs indicate percentages of autophagy flux in HGSOC cells during specific inhibition of SLC1A5 activity (GNPA, 5 mmol/L) and manipulation of claudin-4 expression. **D,** Graphs indicate percentages of autophagy flux in HGSOC cells during specific inhibition of LAT1 (BCH, 25 mmol/L). (Two-tailed Unpaired *t* test and Mann–Whitney test; Kruskal–Wallis test with Dunn’s multiple comparisons; One way ANOVA and Tukey’s multiple comparison test; three independent experiments; significance, *P* < 0.05). Graphs show mean and SEM, scale bar, 20 µm.

To get more insights into this possibility, we evaluated the intracellular distribution of SLC1A5 during the inhibition of autophagy using CQ or its activation using rapamycin. The employment of CQ altered the intracellular distribution of SLC1A5 in all EOC cells. In OVCAR8 cells, we observed a clear accumulation of big vacuoles that excluded SLC1A5 and a similar phenotype in OVCAR3 cells, albeit with less magnitude (Fig. 6A). In OVCA429 cells, we observed the loss of the characteristic accumulation of SLC1A5 during claudin-4 downregulation (Fig. 5E, middle) or L-glutamine limitation (Fig. 5F, middle)). As the effect of CQ in blocking autophagy is through affecting vesicular trafficking, our results suggest that the association of claudin-4 with the intracellular localization of SLC1A5 could be through vesicular trafficking. In contrast, the use of rapamycin resulted in the accumulation of SLC1A5 in large aggregates in OVCAR8 claudin-4 cells, with a similar but reduced phenotype observed in OVCA429 WT cells (Fig. 6B). Together, the inhibition of autophagy or its activation led to changes in the intracellular distribution of SLC1A5, supporting its involvement in the regulation of autophagy in ovarian tumor cells. To specifically determine if SLC1A5 and LAT1 regulate autophagy in EOC cells, we inhibited the transporter function of both proteins as previously described (54). We used GPNA (25 mmol/L) to inhibit SLC1A5 and BCH (5 mmol/L) to inhibit LAT1. Subsequently, we evaluated autophagy flux. We observed that inhibition of both SLC1A5 and LAT1 resulted in increased autophagy activity in all EOC cells (OVCAR8, OVCA429, and OVCAR3), especially with LAT1 inhibition showing particularly significant effects (Fig. 6C and D). These findings imply the functional involvement of these transporters in autophagy regulation in ovarian tumor cells.

Specifically, inhibition of SLC1A5 was associated with a significant increase in autophagy only in OVCAR8 claudin-4 cells at 48 hours, in OVCA429 WT cells at 24 hours, and in OVCAR3 claudin-4 KD cells at 24 and 48 hours. Thus, the amino acid transporter function of SLC1A5 was required to regulate autophagy in these cells negatively (Fig. 6C), showing a differential effect depending on the acute (24 hours) and chronic setting (48 hours) as well as claudin-4 expression. For example, considering the specific inhibition of SLC1A5 along with the effect of claudin-4 manipulation (overexpression and downregulation) on autophagy, which resulted in increased autophagy flux at 24 hours (Fig. 2A–C), the activity of this amino acid transporter seemed to be associated with the upregulation of autophagy during claudin-4 overexpression (Fig. 2A) because such upregulation was significantly reduced in both the acute and chronic setting due to inhibition of SLC1A5. Consequently, autophagy activity was similar between OVCAR8 WT and OVCAR8 claudin-4-overexpressing cells (Fig. 6C, left), suggesting an association between the function of claudin-4 and SLC1A5 in autophagy regulation.

An important question arises regarding the connection between claudin-4 and SLC1A5 in autophagy regulation. When claudin-4 was downregulated in OVCA429 cells, it exhibited behavior similar to WT cells (Fig. 6C, middle) but not in OVCAR3 claudin-4 KD cells, where inhibition of SLC1A5 led to significant increases in autophagy at both 24 and 48 hours (Fig. 6C, right). These differences suggest cell-type-specific effects, possibly influenced by mutations in *TP53* gene (1), known to be involved in autophagy regulation (48, 49). For instance, OVCA429 cells harbor a missense mutation of TP53, while OVCAR3 cells exhibit a *TP53* deep deletion (Fig. 1A; ref. 1). Additionally, SLC1A5 is known to transport L-glutamine, which negatively regulates autophagy by internalizing essential amino acids (Fig. 5D; ref. 54). L-glutamine is transported from the extracellular environment to the cytoplasm, increasing its basal concentration (54), and from lysosomes, which store L-glutamine and other amino acids, to the cytoplasm. This implies that this transport and storage timing is important for regulating autophagy (57). Hence, inhibiting SLC1A5 would reduce L-glutamine availability within the cytoplasm, either coming from the extracellular milieu or lysosomes, potentially in a time-dependent manner. This is supported by the observation that inhibition of SLC1A5 had a more pronounced effect in the chronic setting than in the acute setting regarding autophagy activation (Fig. 6C). Furthermore, SLC1A5 and LAT1 form a cyclic and bidirectional system to transport essential amino acids, which regulates upstream autophagy (54). In this system, L-glutamine plays a key role through its influx and efflux, with SLC1A5 internalizing this amino acid as LAT1 exports it while internalizing essential amino acids (54). In this context, inhibition of SLC1A5 would alter this bidirectional system and, consequently, autophagy (54). In supporting this notion, inhibition of LAT1, which promotes the internalization of essential amino acids (54), resulted in a more pronounced upregulation of autophagy in EOC cells. This highlights a more significant effect of LAT1 activity on autophagy regulation compared to SLC1A5, especially at earlier stages of cell culture, emphasizing the timing for the internalization of amino acids mediated by LAT1 (Fig. 6D). Considering the inhibition of LAT1 alongside claudin-4 manipulation, we observed a similar outcome to that seen with SLC1A5 inhibition regarding autophagy activation during claudin-4 modulation (overexpression and downregulation; Fig. 2A–C). This suggests a potential relationship between claudin-4 and LAT1 function in autophagy regulation (Fig. 6D, left), highlighting previously noted cell type–specific genetic differences (Fig. 1A; ref. 1) between OVCA429 (Fig. 6D, middle) and OVCAR3 cells (Fig. 6D, right). Notably, inhibiting both SLC1A5 and LAT1 in OVCAR3 cells during claudin-4 downregulation resulted in a more significant effect on autophagy, implying a potentially larger role for the proposed bidirectional transport system (54). In summary, the specific inhibition of SLC1A5 and LAT1 disrupted autophagy in EOC cells and altered the impact of claudin-4 expression on autophagy regulation. This strongly suggests a functional link between amino acid transport mediated by SLC1A5 and LAT1 and claudin-4 in EOC cells, where claudin-4 may support the transport activity of SLC1A5 and LAT1.

### The claudin-4 expression modifies the expression and function of amino acid transporters, SLC1A5 and LAT1

**Figure 7:**
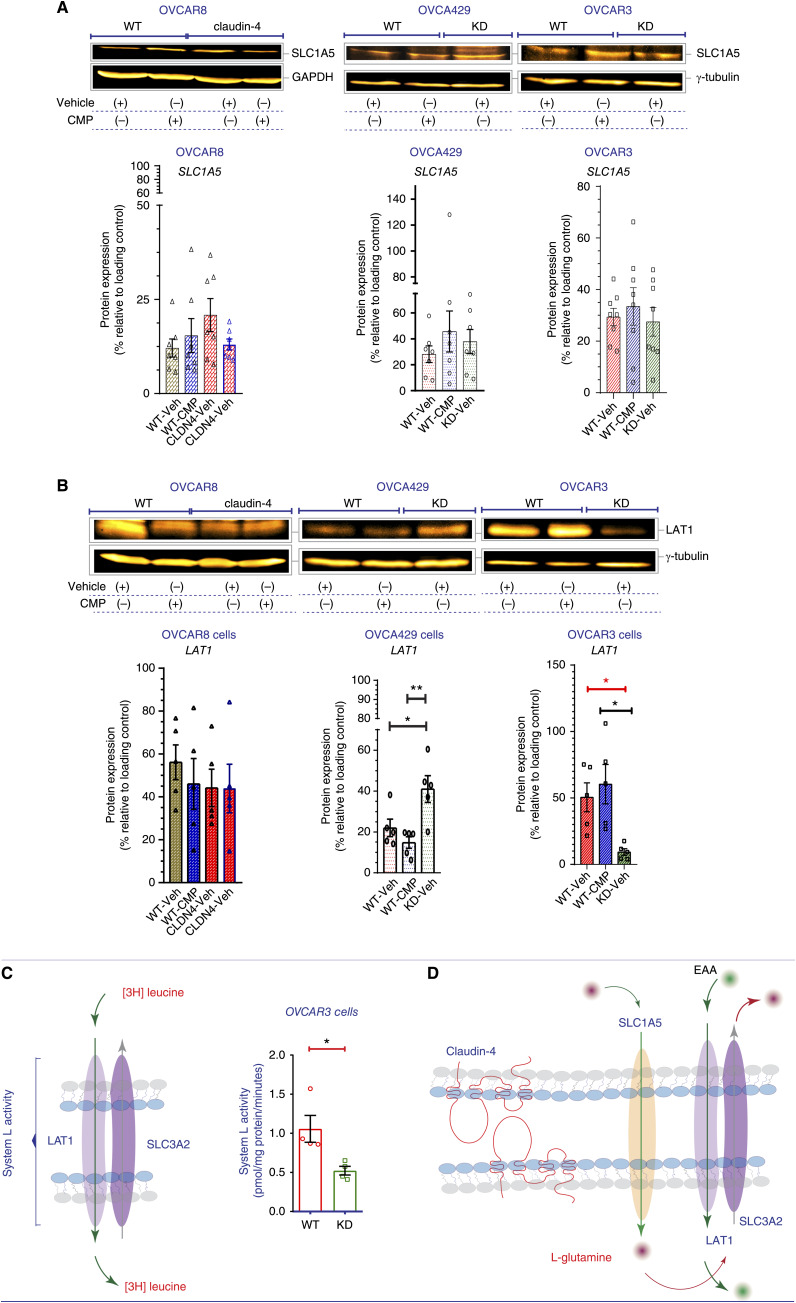
Impact of claudin-4 in the expression of transporters of amino acids and the uptake of essential amino acids in HGSOC cells. The effect of claudin-4 manipulation (overexpression and downregulation) in the expression of SLC1A5 and LAT1 was measured via immunoblotting in HGSOC cells (OVCAR8, OVCA429, and OVCAR3). Direct amino acid uptake (essential amino acids, EAA; [3H]leucine) was analyzed in OVCAR3 cells (culture for 24 hours). **A** and **B,** Top, show representative immunoblots for SLC1A5 and LAT1 in HGSOC cells during claudin-4 disruption, respectively; (bottom), corresponding quantification (four independent experiments). **C,** Left, illustration highlighting a heterodimer formed by LAT and SLC3A2 which internalizes essential amino acids; right, uptake measurement of the essential amino acid leucine ([3H]leucine; three independent experiments; Two-tail *t* test). **D,** Model [adapted from reference: (54)] proposing an axis formed by claudin-4, SLC1A5, and LAT1 which could regulate autophagy in HGSOC cells. In this model, claudin-4 is contributing to determine the intracellular localization of theses transporters of amino acids, and possibly stabilizing their function in membranes. (significance, *P* < 0.05). Graphs show mean and SEM.

We analyzed the protein expression of SLC1A5 and LAT1 using immunoblotting and found changes in both amino acid transporters. Specifically, during claudin-4 overexpression (Fig. 7A), we observed an increase in SLC1A5 expression. However, the changes in LAT1 protein levels varied significantly among EOC cells, particularly in OVCA429 and in OVCAR3 claudin-4 KD cells. In OVCAR8 claudin-4-overexpressing cells, there was a trend of reduced LAT1 expression (Fig. 7B, left). Conversely, OVCA429 claudin-4 KD cells showed a significant increase in LAT1 expression (Fig. 7B, middle), while OVCAR3 claudin-4 KD cells exhibited significantly lower LAT1 expression (Fig. 7B, right). These differences underscore the importance of claudin-4’s association with the bidirectional transport system (SLC1A5/LAT1) that regulates autophagy (54), as well as certain genetic alterations (Fig. 1A; ref. 1). Furthermore, the decrease in LAT1 in OVCAR3 cells during claudin-4 downregulation (Fig. 7B, right) prompted us to investigate claudin-4’s direct effect on amino acid uptake. To confirm this effect, we directly measured the system L activity (mediated by LAT1 to internalize essential amino acids, EAA) using radioactive [3H] leucine (Fig. 7C), as previously reported (26). The results showed that claudin-4 downregulation was associated with a significant reduction in the internalization of essential amino acids in EOC cells, as indicated by the uptake of [3H] leucine (Fig. 7C). In summary, our data demonstrate that claudin-4 influences the function of amino acid transporters, specifically SLC1A5 and LAT1 (54). This mechanism likely involves claudin-4’s contribution to the proper localization and function of SLC1A5 and LAT1, which in turn regulate autophagy, aiding in the clearance of genetic aberrations (Fig. 7D).

## Discussion

Genome instability is a hallmark of cancer, indicating that tumor cells possess cellular mechanisms to tolerate high levels of genetic aberrations (10–12). However, it is not well understood how tumor cells limit the accumulation of such modifications to avoid catastrophic chromosomic alterations and cell death. Here, we demonstrate that claudin-4, traditionally described as a TJ protein, participates in a tolerance mechanism for genomic instability in ovarian tumor cells. This mechanism limits the accumulation of a form of genomic instability, micronuclei, through autophagy. Thus, this mechanism contributes to increasing the threshold tolerance for genomic instability by ovarian tumor cells.

In this study, we showed that micronuclei are a common form of genomic instability in EOC cells (OVCAR8, OVCA429, and OVCAR3 cells), which can potentially collapse due to the absence of components of the nuclear envelope (lamin B1 and lamin A/C), partly associated with *TP53* mutations (Fig. 1A; ref. 1). An expected consequence of micronuclei collapse is the release of DNA into the cytoplasm, which can be detected by cGAS, thus activating the cGAS-STING signaling pathway (42, 43). Given that claudin-4 is related to micronuclei formation, possibly through its role in regulating the cell cycle, as previously reported (23), and suggested by our metabolomics data (see Supplementary Fig. S3A), claudin-4 is also anticipated to be involved in micronuclei generation and collapse. This hypothesis is supported by findings indicating that the cGAS-STING signaling is typically inhibited in ovarian tumor cells (44), suggesting a mechanism to clear collapsing micronuclei and prevent DNA released into the cytoplasm. In this context, autophagy emerges as a key cellular process, as it is reported to prioritize the clearance of cytosolic DNA, a fundamental function of the cGAS-STING signaling (45). Furthermore, this cellular process has been shown to remove micronuclei in tumor cells (46).

The role of claudin-4 in autophagy was demonstrated using the tandem GFP-mCherry-LC3 through flow cytometry and confocal microscopy, and this cellular process was associated with engulfing micronuclei, highlighting the role of autophagy in genomic instability (52, 60). Claudin-4 overexpression appeared to act as a positive regulator of autophagy, as evidenced by the correlation between its overexpression and sustained increase autophagy (Fig. 2A). Conversely, its downregulation did not show a similar trend, indicating a dominant effect other than a decrease in claudin-4 expression that upregulated autophagy during extended cultured conditions (24 vs. 48 hours). However, the association of claudin-4 with autophagy was found to be related to the activity of transporters of amino acids, SLC1A5 and LAT1. Given that a well-known characteristic of cell culture over time is the decrease of nutrient availability, including amino acids (50), these results suggested that the effect of claudin-4 on autophagy is related to transport of amino acids mediated by SLC1A5 and LAT1. This implication is supported by the regulation of autophagy, which is associated with amino acid availability (61), as well as by the reported regulation of autophagy through these amino acid transporters and mTOR (54). Furthermore, the negative regulator of autophagy and sensor of amino acids, mTOR (51) appeared to be inhibited during claudin-4 downregulation.

Furthermore, global metabolomics analysis indicated that regulation of amino acid transport was one of the key cellular processes altered due to claudin-4 downregulation, correlating with a reported bidirectional transport system formed by SLC1A5 and LAT1/SLC3A2 (54), which, in turn, are claudin-4-interacting proteins (39). Importantly, this bidirectional system regulates autophagy upstream of mTOR (54). Additionally, SLC1A5 and LAT1 colocalized with claudin-4 and their intracellular distribution was disrupted via claudin-4 modulation. Also, SLC1A5 and LAT1 inhibition resulted in autophagy upregulation in different ovarian tumor cells (Fig. 6C and D), suggesting an association of claudin-4 with these amino acid transporters as a potential element regulating autophagy. Interestingly, the role of claudin-4 in autophagy and genomic instability, as well as its association with amino acid transporters, strongly suggest a novel and unique clinical significance of claudin-4 in multiple cancer types (Fig. 4D).

Consequently, the existence of a claudin-4/SLC1A5/LAT1 biological axis in HGSOC cells has the potential to be a targetable mechanism that facilitates the development of therapy resistance. For example, claudin-4 is associated with developing therapy resistance independently (2), as are LAT1 (62) and SLC1A5.(63) Additionally, a key phenotype during ovarian cancer development is ascites formation, which is a hallmark of ovarian cancer malignancy and is closely related to the availability of metabolites for tumor cells (64–67), which can also negatively modulate immune cells (68). Thus, targeting this proposed axis has the potential to inhibit tumor growth and prevent development of therapy resistance. For example, CMP can reduce tumor growth *in vivo* by potentially inducing apoptosis in tumor cells by interfering with claudin-4 (17), while the expression of LAT1 has been reported to be modified using forskolin (69), thus potentially altering the activity of the bidirectional transport system of amino acids (54). Moreover, it is known that the availability of specific amino acids is a determinant in inducing or arresting tumor growth (70). Together, our data highlights a novel autophagy regulatory axis mediated via claudin-4’s interaction with amino acid transporters that aids in the clearance of micronuclei.

## Supplementary Material

Supplementary Table 1 BioID Data - Claudin-4 proximal proteins

Supplementary Figure 1 Confirmation of claudin-4 expression and modulation of the autophagy pathway

Supplementary Table 2 Metabolomics OVCA429 and OVCAR3

Supplementary Figure 2 Gating strategy for autophagic flux

Supplementary Table 3 Survival statistics of claudin-4 with its interacting partners.

Supplementary Figure 3 Metabolic enrichments

Supplementary Table 4 Survival statistics of claudin-4-interacting partners.

Supplementary Figure 4 Survival analysis in breast, lung, and stomach cancers.

Movie 1 Movie 1 - Hoechst

Movie 2 Movie 2 - GFP

Movie 3 Movie 3 - mCherry

Movie 4 Movie 4 - Merge
