# Supplementary material for: Claudin-4 Modulates Autophagy via SLC1A5/LAT1 as a Mechanism to Regulate Micronuclei
Source: Cancer Res Commun. 2024 Jul 2;4(7):1625–42. doi: 10.1158/2767-9764.CRC-24-0240 (PMC11218812; doi:10.1158/2767-9764.CRC-24-0240)
Supplement: Supplementary Table 1 — BioID Data - Claudin-4 proximal proteins [file crc-24-0240_supplementary_table_1_suppst1.docx]

| **Supplemental Table 1** | |  |  |
| --- | --- | --- | --- |
| ***Alternate ID*** | ***Control sample 1*** | ***CLDN4 sample 2*** | ***FC Log 2 (Claudin-4/Control)*** |
| TENM2 | 4 | 405 | 6.661778 |
| SLC3A2 | 16 | 182 | 3.507795 |
| EPHA2 | 19 | 204 | 3.424498 |
| MCAM | 4 | 215 | 5.748193 |
| NOTCH2 | 21 | 162 | 2.947533 |
| CSPG4 | 1 | 156 | 7.285402 |
| KIRREL1 | 1 | 77 | 6.266787 |
| ITGB1 | 5 | 226 | 5.498251 |
| PLCB4 | 15 | 122 | 3.023847 |
| LEMD3 | 4 | 57 | 3.83289 |
| NECTIN2 | 1 | 51 | 5.672425 |
| ROBO1 | 8 | 90 | 3.491853 |
| EPHB2 | 1 | 38 | 5.247928 |
| RAB7A | 7 | 50 | 2.836501 |
| ICAM1 | 1 | 41 | 5.357552 |
| CXADR | 1 | 55 | 5.78136 |
| SLC1A5 | 6 | 52 | 3.115477 |
| JAG2 | 1 | 65 | 6.022368 |
| PREB | 5 | 51 | 3.350497 |
| SLITRK4 | 3 | 85 | 4.824428 |
| YKT6 | 8 | 154 | 4.266787 |
| ACACB | 3 | 57 | 4.247928 |
| EFNB1 | 2 | 21 | 3.392317 |
| TAOK3 | 9 | 72 | 3 |
| CD44 | 7 | 31 | 2.146841 |
| SPTBN2 | 1 | 104 | 6.70044 |
| ITGA6 | 1 | 42 | 5.392317 |
| MARK3 | 1 | 38 | 5.247928 |
| AP1B1 | 1 | 28 | 4.807355 |
| IGF2R | 29 | 83 | 1.517058 |
| SPTBN1 | 35 | 99 | 1.500074 |
| MARCKS | 1 | 17 | 4.087463 |
| AP2B1 | 22 | 75 | 1.769387 |
| FLRT2 | 1 | 50 | 5.643856 |
| SLC39A10 | 1 | 32 | 5 |
| MARK2 | 1 | 18 | 4.169925 |
| BAIAP2 | 7 | 37 | 2.402098 |
| ELFN1 | 1 | 37 | 5.209453 |
| SLC6A8 | 1 | 20 | 4.321928 |
| BSG | 1 | 17 | 4.087463 |
| ZDHHC5 | 4 | 51 | 3.672425 |
| HMOX2 | 3 | 32 | 3.415037 |
| DSG2 | 18 | 184 | 3.353637 |
| CCDC47 | 1 | 37 | 5.209453 |
| NOTCH1 | 1 | 30 | 4.906891 |
| EFNB2 | 1 | 19 | 4.247928 |
| VANGL1 | 1 | 18 | 4.169925 |
| ITGB5 | 1 | 49 | 5.61471 |
| ABCC5 | 1 | 34 | 5.087463 |
| SLC20A2 | 1 | 16 | 4 |
| CDC42EP4 | 2 | 25 | 3.643856 |
| CDC42BPA | 3 | 32 | 3.415037 |
| INPP4B | 8 | 65 | 3.022368 |
| SLITRK5 | 1 | 55 | 5.78136 |
| IGSF3 | 1 | 51 | 5.672425 |
| LNPEP | 1 | 49 | 5.61471 |
| CPD | 1 | 34 | 5.087463 |
| ATP2B1 | 1 | 26 | 4.70044 |
| SLC4A7 | 13 | 99 | 2.928917 |
| ADD3 | 5 | 27 | 2.432959 |
| MYOF | 12 | 31 | 1.369234 |
| EGFR | 46 | 133 | 1.53172 |
| SEMA4C | 1 | 43 | 5.426265 |
| TMEM2 | 1 | 42 | 5.392317 |
| PANX1 | 1 | 31 | 4.954196 |
| ADAM9 | 1 | 23 | 4.523562 |
| CLCC1 | 3 | 37 | 3.624491 |
| ARL13B | 4 | 30 | 2.906891 |
| LLGL1 | 15 | 48 | 1.678072 |
| ABCC1 | 1 | 54 | 5.754888 |
| LYN | 3 | 34 | 3.5025 |
| YES1 | 5 | 35 | 2.807355 |
| CDCA3 | 4 | 11 | 1.459432 |
| VAMP3 | 6 | 14 | 1.222392 |
| FERMT2 | 26 | 133 | 2.354843 |
| CTNND1 | 51 | 215 | 2.075768 |
| BASP1 | 11 | 46 | 2.06413 |
| EFR3A | 11 | 52 | 2.241008 |
| CAVIN1 | 3 | 16 | 2.415037 |
| ESYT1 | 47 | 106 | 1.173332 |
| TRIP11 | 27 | 128 | 2.245112 |
| PEAK1 | 11 | 30 | 1.447459 |
| PGRMC2 | 19 | 81 | 2.091922 |
| SNAP23 | 8 | 49 | 2.61471 |
| ROCK2 | 6 | 30 | 2.321928 |
| EPB41L5 | 20 | 55 | 1.459432 |
| DLG1 | 18 | 48 | 1.415037 |
| ATP1A1 | 20 | 83 | 2.053111 |
| GOLGB1 | 36 | 194 | 2.429988 |
| AKAP12 | 101 | 501 | 2.310455 |
| PHACTR4 | 36 | 78 | 1.115477 |
| PCCB | 7 | 48 | 2.777608 |
| EPB41L2 | 49 | 138 | 1.493815 |
| ACACA | 72 | 200 | 1.473931 |
| MCCC1 | 154 | 692 | 2.167842 |
| PCCA | 127 | 832 | 2.711755 |
| EHBP1 | 21 | 51 | 1.280108 |
| TOR1AIP1 | 7 | 22 | 1.652077 |
| TMPO | 42 | 187 | 2.154577 |
| KRT2 | 132 | 349 | 1.402689 |
| AHNAK | 2122 | 7542 | 1.829522 |
| ZC3HAV1 | 29 | 59 | 1.024662 |
| AHNAK2 | 156 | 533 | 1.77259 |
| TTF1 | 11 | 31 | 1.494765 |
| CCDC88A | 25 | 52 | 1.056584 |
| SNAP29 | 7 | 24 | 1.777608 |
| KRT10 | 208 | 860 | 2.047753 |
| UTRN | 22 | 75 | 1.769387 |
| PTPN1 | 19 | 37 | 0.961526 |
| CKAP4 | 27 | 50 | 0.888969 |
| KRT5 | 26 | 48 | 0.884523 |
| JUP | 29 | 52 | 0.842459 |
| EPB41 | 14 | 24 | 0.777608 |
| PC | 80 | 137 | 0.776104 |
| SOWAHC | 10 | 17 | 0.765535 |
| FAM91A1 | 26 | 43 | 0.725825 |
| ERBIN | 119 | 186 | 0.644341 |
| SCRIB | 72 | 111 | 0.624491 |
| PKN2 | 18 | 27 | 0.584963 |
| EHD1 | 24 | 33 | 0.459432 |
| NUMB | 33 | 44 | 0.415037 |
| TMPO | 83 | 96 | 0.209923 |
| FAU | 21 | 24 | 0.192645 |
| KRT14 | 30 | 53 | 0.82103 |
| VAPA | 18 | 31 | 0.784271 |
| RAI14 | 64 | 108 | 0.754888 |
| STX5 | 17 | 28 | 0.719892 |
| KRT1 | 240 | 388 | 0.693022 |
| RRBP1 | 32 | 50 | 0.643856 |
| RPS27A | 34 | 52 | 0.612977 |
| HSPA1A | 66 | 93 | 0.494765 |
| KTN1 | 98 | 137 | 0.483322 |
| H1F0 | 87 | 120 | 0.463947 |
| NOP56 | 107 | 147 | 0.458205 |
| RAB11FIP1 | 21 | 28 | 0.415037 |
| TACC1 | 34 | 45 | 0.40439 |
| EPB41L3 | 84 | 111 | 0.402098 |
| SLC25A3 | 10 | 13 | 0.378512 |
| GOLGA5 | 23 | 28 | 0.283793 |
| KNOP1 | 31 | 37 | 0.255257 |
| MTDH | 37 | 44 | 0.249978 |
| RAB11FIP5 | 19 | 21 | 0.14439 |
| GPRIN1 | 80 | 87 | 0.121015 |
| AFDN | 154 | 164 | 0.090765 |
| RDX | 28 | 29 | 0.050626 |
| ALCAM | 1 | 75 | 6.228819 |
| USP32 | 1 | 55 | 5.78136 |
| NECTIN3 | 1 | 47 | 5.554589 |
| PTPRF | 1 | 40 | 5.321928 |
| SCARB1 | 1 | 39 | 5.285402 |
| MRC2 | 1 | 37 | 5.209453 |
| WDR20 | 1 | 37 | 5.209453 |
| TMEM87A | 1 | 36 | 5.169925 |
| TFRC | 1 | 36 | 5.169925 |
| DIP2A | 1 | 35 | 5.129283 |
| SLC12A2 | 1 | 33 | 5.044394 |
| DHRS7 | 1 | 31 | 4.954196 |
| OCLN | 1 | 30 | 4.906891 |
| TRPM4 | 1 | 25 | 4.643856 |
| TENM3 | 1 | 24 | 4.584963 |
| IGF1R | 1 | 24 | 4.584963 |
| CLGN | 1 | 23 | 4.523562 |
| SPEG | 1 | 23 | 4.523562 |
| SPECC1 | 1 | 23 | 4.523562 |
| FAM135A | 1 | 23 | 4.523562 |
| STEAP3 | 1 | 23 | 4.523562 |
| DSC2 | 1 | 22 | 4.459432 |
| ADCY9 | 1 | 22 | 4.459432 |
| LSR | 1 | 22 | 4.459432 |
| LMAN1 | 1 | 21 | 4.392317 |
| PLXNA1 | 1 | 21 | 4.392317 |
| ITM2B | 1 | 21 | 4.392317 |
| CLDN4 | 1 | 21 | 4.392317 |
| CBARP | 1 | 21 | 4.392317 |
| RELL1 | 1 | 20 | 4.321928 |
| LRP8 | 1 | 20 | 4.321928 |
| ROR2 | 1 | 20 | 4.321928 |
| SLC7A11 | 1 | 20 | 4.321928 |
| PPFIBP1 | 3 | 58 | 4.273018 |
| ECE1 | 1 | 19 | 4.247928 |
| FMNL1 | 1 | 19 | 4.247928 |
| PCDHGB5 | 1 | 19 | 4.247928 |
| CNTNAP1 | 1 | 19 | 4.247928 |
| FAM171B | 1 | 19 | 4.247928 |
| EIF2AK3 | 1 | 19 | 4.247928 |
| STX11 | 1 | 19 | 4.247928 |
| PTPRJ | 1 | 18 | 4.169925 |
| LAT2 | 1 | 18 | 4.169925 |
| SCAMP1 | 1 | 18 | 4.169925 |
| PLXNB2 | 1 | 18 | 4.169925 |
| FGD6 | 1 | 17 | 4.087463 |
| INSR | 1 | 17 | 4.087463 |
| SLC19A1 | 1 | 17 | 4.087463 |
| GOPC | 1 | 17 | 4.087463 |
| BAIAP2L1 | 1 | 16 | 4 |
| PTPRM | 1 | 16 | 4 |
| RALGAPA1 | 1 | 16 | 4 |
| TMEM209 | 1 | 16 | 4 |
| EPB41L1 | 1 | 16 | 4 |
| DSC1 | 1 | 16 | 4 |
| NEO1 | 1 | 16 | 4 |
| FAM171A1 | 1 | 16 | 4 |
| THSD1 | 2 | 32 | 4 |
| KIAA0754 | 1 | 15 | 3.906891 |
| PKP4 | 1 | 15 | 3.906891 |
| DCBLD1 | 1 | 15 | 3.906891 |
| EPHB1 | 1 | 15 | 3.906891 |
| ITM2C | 1 | 15 | 3.906891 |
| PTK7 | 2 | 29 | 3.857981 |
| IL6ST | 1 | 14 | 3.807355 |
| DLG5 | 1 | 14 | 3.807355 |
| FAM83B | 1 | 14 | 3.807355 |
| PLSCR3 | 1 | 14 | 3.807355 |
| DCBLD2 | 1 | 14 | 3.807355 |
| PPP1R16B | 1 | 14 | 3.807355 |
| UBIAD1 | 1 | 14 | 3.807355 |
| LRRC8C | 1 | 14 | 3.807355 |
| TRPM7 | 1 | 13 | 3.70044 |
| EPHB4 | 1 | 13 | 3.70044 |
| RALGAPA2 | 1 | 13 | 3.70044 |
| TLDC1 | 1 | 13 | 3.70044 |
| PLCL2 | 1 | 13 | 3.70044 |
| SLC6A15 | 1 | 13 | 3.70044 |
| VEZT | 1 | 13 | 3.70044 |
| SLC38A2 | 1 | 13 | 3.70044 |
| CLMP | 1 | 13 | 3.70044 |
| ANKRD26 | 1 | 12 | 3.584963 |
| APBB2 | 1 | 12 | 3.584963 |
| TMEM199 | 1 | 12 | 3.584963 |
| PPFIA1 | 1 | 12 | 3.584963 |
| FAM171A2 | 1 | 12 | 3.584963 |
| KIAA1522 | 1 | 12 | 3.584963 |
| ARG1 | 1 | 12 | 3.584963 |
| FAM234A | 1 | 12 | 3.584963 |
| SLC38A5 | 1 | 12 | 3.584963 |
| SLC5A3 | 1 | 12 | 3.584963 |
| F3 | 1 | 12 | 3.584963 |
| LAYN | 1 | 12 | 3.584963 |
| TRIM13 | 2 | 24 | 3.584963 |
| ADGRL2 | 4 | 46 | 3.523562 |
| STT3B | 1 | 11 | 3.459432 |
| RAB23 | 1 | 11 | 3.459432 |
| SLC16A3 | 1 | 11 | 3.459432 |
| FARP1 | 1 | 11 | 3.459432 |
| PODXL | 1 | 11 | 3.459432 |
| USP43 | 1 | 11 | 3.459432 |
| SLC39A6 | 1 | 11 | 3.459432 |
| TBC1D10A | 1 | 11 | 3.459432 |
| SHB | 1 | 11 | 3.459432 |
| LAMTOR1 | 1 | 11 | 3.459432 |
| CDC42EP1 | 2 | 21 | 3.392317 |
| RAB3IL1 | 1 | 10 | 3.321928 |
| SYTL4 | 1 | 10 | 3.321928 |
| PGRMC1 | 1 | 10 | 3.321928 |
| ZDHHC8 | 1 | 10 | 3.321928 |
| VRK2 | 1 | 10 | 3.321928 |
| DAG1 | 1 | 10 | 3.321928 |
| LZTS2 | 1 | 10 | 3.321928 |
| DDRGK1 | 1 | 10 | 3.321928 |
| CANT1 | 1 | 10 | 3.321928 |
| SLC29A1 | 1 | 10 | 3.321928 |
| ROCK1 | 5 | 49 | 3.292782 |
| ELFN2 | 1 | 9 | 3.169925 |
| PLCB1 | 1 | 9 | 3.169925 |
| ELMOD3 | 1 | 9 | 3.169925 |
| DNAJC1 | 1 | 9 | 3.169925 |
| RAB6A | 1 | 9 | 3.169925 |
| RP2 | 1 | 9 | 3.169925 |
| GGT7 | 1 | 9 | 3.169925 |
| LRRC57 | 1 | 9 | 3.169925 |
| LRRC8A | 1 | 9 | 3.169925 |
| ARHGAP39 | 1 | 9 | 3.169925 |
| CERS4 | 1 | 9 | 3.169925 |
| CLDN1 | 1 | 9 | 3.169925 |
| CASP14 | 1 | 9 | 3.169925 |
| CD320 | 1 | 9 | 3.169925 |
| SLC9A1 | 1 | 9 | 3.169925 |
| TBC1D22B | 1 | 9 | 3.169925 |
| POR | 2 | 18 | 3.169925 |
| STK10 | 7 | 62 | 3.146841 |
| SRC | 3 | 26 | 3.115477 |
| SLC16A1 | 4 | 34 | 3.087463 |
| PARD3 | 2 | 16 | 3 |
| ZFPL1 | 2 | 14 | 2.807355 |
| GORASP2 | 4 | 28 | 2.807355 |
| FLOT1 | 2 | 13 | 2.70044 |
| SYNE2 | 3 | 19 | 2.662965 |
| NDRG1 | 3 | 18 | 2.584963 |
| UBXN4 | 3 | 17 | 2.5025 |
| CDKAL1 | 5 | 26 | 2.378512 |
| GOLGA3 | 2 | 10 | 2.321928 |
| GPAT4 | 2 | 10 | 2.321928 |
| NDRG3 | 2 | 10 | 2.321928 |
| TOR1AIP2 | 3 | 15 | 2.321928 |
| FMN2 | 6 | 29 | 2.273018 |
| GNA13 | 5 | 24 | 2.263034 |
| EMD | 5 | 24 | 2.263034 |
| PDXDC1 | 4 | 19 | 2.247928 |
| TAOK1 | 4 | 19 | 2.247928 |
| CLCN7 | 3 | 14 | 2.222392 |
| PACSIN2 | 3 | 14 | 2.222392 |
| TMX1 | 3 | 14 | 2.222392 |
| KRT78 | 5 | 22 | 2.137504 |
| ARHGAP21 | 3 | 13 | 2.115477 |
| CISD2 | 3 | 13 | 2.115477 |
| SLC7A5 | 3 | 13 | 2.115477 |
| FLG2 | 6 | 25 | 2.058894 |
| SCYL3 | 2 | 8 | 2 |
| MAGI1 | 5 | 19 | 1.925999 |
| ACBD3 | 7 | 26 | 1.893085 |
| FLG | 3 | 11 | 1.874469 |
| RASAL2 | 7 | 25 | 1.836501 |
| DSG1 | 9 | 32 | 1.830075 |
| PACS1 | 4 | 14 | 1.807355 |
| LBR | 6 | 21 | 1.807355 |
| KRT77 | 6 | 21 | 1.807355 |
| PALM | 3 | 10 | 1.736966 |
| OSBPL8 | 9 | 30 | 1.736966 |
| RFTN1 | 7 | 23 | 1.716207 |
| CNP | 6 | 19 | 1.662965 |
| OSBPL11 | 3 | 9 | 1.584963 |
| RPN2 | 3 | 9 | 1.584963 |
| ABI1 | 3 | 9 | 1.584963 |
| SPAG1 | 4 | 12 | 1.584963 |
| PLCB3 | 5 | 15 | 1.584963 |
| UBE2J1 | 7 | 20 | 1.514573 |
| RABL3 | 4 | 11 | 1.459432 |
| SRPRA | 7 | 19 | 1.440573 |
| KPRP | 7 | 19 | 1.440573 |
| ARHGAP1 | 7 | 19 | 1.440573 |
| SMPD4 | 3 | 8 | 1.415037 |
| STBD1 | 6 | 16 | 1.415037 |
| APBB1 | 5 | 13 | 1.378512 |
| TSC1 | 10 | 26 | 1.378512 |
| SLK | 26 | 66 | 1.343954 |
| HLA-A | 4 | 10 | 1.321928 |
| PTPN2 | 7 | 17 | 1.280108 |
| ESYT2 | 8 | 19 | 1.247928 |
| JPH1 | 3 | 7 | 1.222392 |
| PALM2 | 3 | 7 | 1.222392 |
| SNX3 | 4 | 9 | 1.169925 |
| MAP4K4 | 14 | 31 | 1.146841 |
| SLC38A1 | 6 | 13 | 1.115477 |
| EXOC3 | 13 | 28 | 1.106915 |
| RAB3B | 7 | 15 | 1.099536 |
| SEC16A | 11 | 23 | 1.06413 |
| PAK4 | 8 | 16 | 1 |
| ALDH3A2 | 9 | 18 | 1 |
| FAM129B | 22 | 44 | 1 |
| SEPT7 | 17 | 33 | 0.956931 |
| PLEKHA5 | 16 | 31 | 0.954196 |
| ANKLE2 | 13 | 25 | 0.943416 |
| LRBA | 10 | 19 | 0.925999 |
| ANKRD50 | 6 | 11 | 0.874469 |
| HIST1H1C | 5 | 9 | 0.847997 |
| WASF2 | 9 | 16 | 0.830075 |
| GAB1 | 4 | 7 | 0.807355 |
| LUC7L3 | 12 | 21 | 0.807355 |
| VAPB | 16 | 28 | 0.807355 |
| VAT1 | 14 | 24 | 0.777608 |
| DIAPH3 | 18 | 30 | 0.736966 |
| FAM129A | 5 | 8 | 0.678072 |
| STAM | 10 | 16 | 0.678072 |
| LLPH | 12 | 19 | 0.662965 |
| LYAR | 31 | 49 | 0.660514 |
| CAV1 | 9 | 14 | 0.63743 |
| SON | 85 | 131 | 0.624032 |
| CHMP4B | 4 | 6 | 0.584963 |
| TANC1 | 6 | 9 | 0.584963 |
| RPL23A | 10 | 15 | 0.584963 |
| HGS | 11 | 16 | 0.540568 |
| SEPT10 | 9 | 13 | 0.530515 |
| NOP58 | 47 | 67 | 0.5115 |
| LRSAM1 | 5 | 7 | 0.485427 |
| SERPINH1 | 5 | 7 | 0.485427 |
| INF2 | 15 | 21 | 0.485427 |
| CARMIL1 | 38 | 53 | 0.479993 |
| KRT17 | 18 | 25 | 0.473931 |
| FCHO2 | 6 | 8 | 0.415037 |
| LMNA | 12 | 16 | 0.415037 |
| GNL3 | 27 | 36 | 0.415037 |
| PICALM | 16 | 21 | 0.392317 |
| STXBP3 | 13 | 17 | 0.387023 |
| PSMC1 | 10 | 13 | 0.378512 |
| DOCK7 | 15 | 19 | 0.341037 |
| CANX | 12 | 15 | 0.321928 |
| HIST1H1B | 20 | 25 | 0.321928 |
| TRIO | 42 | 52 | 0.308122 |
| ARFGEF1 | 9 | 11 | 0.289507 |
| SNX9 | 18 | 22 | 0.289507 |
| ARGLU1 | 19 | 23 | 0.275634 |
| UQCRC1 | 11 | 13 | 0.241008 |
| RPL17 | 11 | 13 | 0.241008 |
| PI4KA | 6 | 7 | 0.222392 |
| RPL29 | 6 | 7 | 0.222392 |
| DST | 93 | 108 | 0.215729 |
| SYAP1 | 13 | 15 | 0.206451 |
| SREK1IP1 | 7 | 8 | 0.192645 |
| HIST1H3A | 14 | 16 | 0.192645 |
| DLC1 | 14 | 16 | 0.192645 |
| VDAC2 | 14 | 16 | 0.192645 |
| TTK | 8 | 9 | 0.169925 |
| HIST1H1D | 24 | 27 | 0.169925 |
| RPL18 | 9 | 10 | 0.152003 |
| LMNB1 | 10 | 11 | 0.137504 |
| TAF7 | 10 | 11 | 0.137504 |
| SEC22B | 10 | 11 | 0.137504 |
| CPNE3 | 11 | 12 | 0.125531 |
| ARHGAP29 | 13 | 14 | 0.106915 |
| MMTAG2 | 13 | 14 | 0.106915 |
| LRRC59 | 17 | 18 | 0.082462 |
