# Supplementary material for: Claudin-4 Modulates Autophagy via SLC1A5/LAT1 as a Mechanism to Regulate Micronuclei
Source: Cancer Res Commun. 2024 Jul 2;4(7):1625–42. doi: 10.1158/2767-9764.CRC-24-0240 (PMC11218812; doi:10.1158/2767-9764.CRC-24-0240)
Supplement: Supplementary Figure 1 — Confirmation of claudin-4 expression and modulation of the autophagy pathway [file crc-24-0240_supplementary_figure_1_suppsf1.docx]

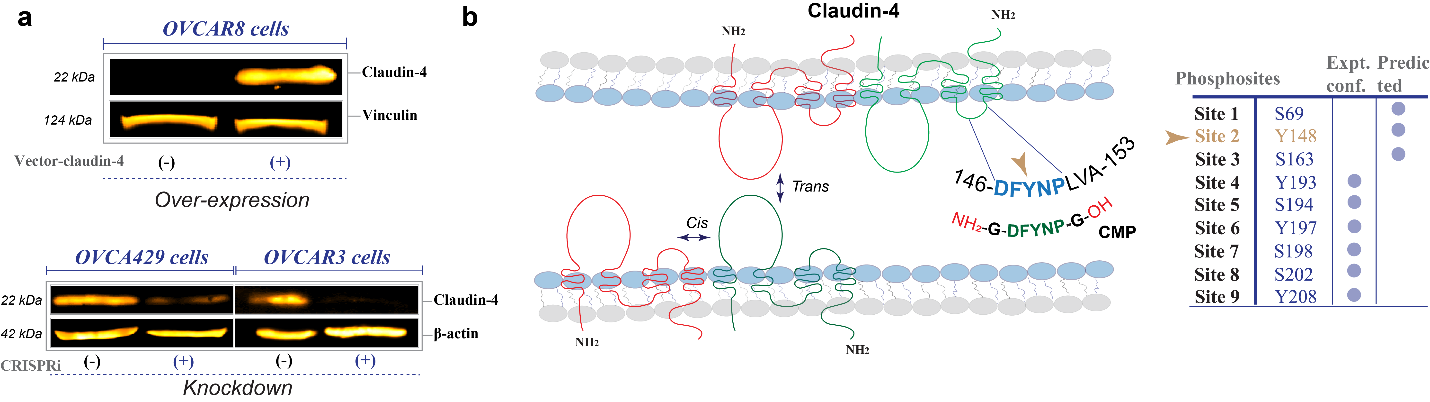
**Supplementary Figure 1, Villagomez, 2024**


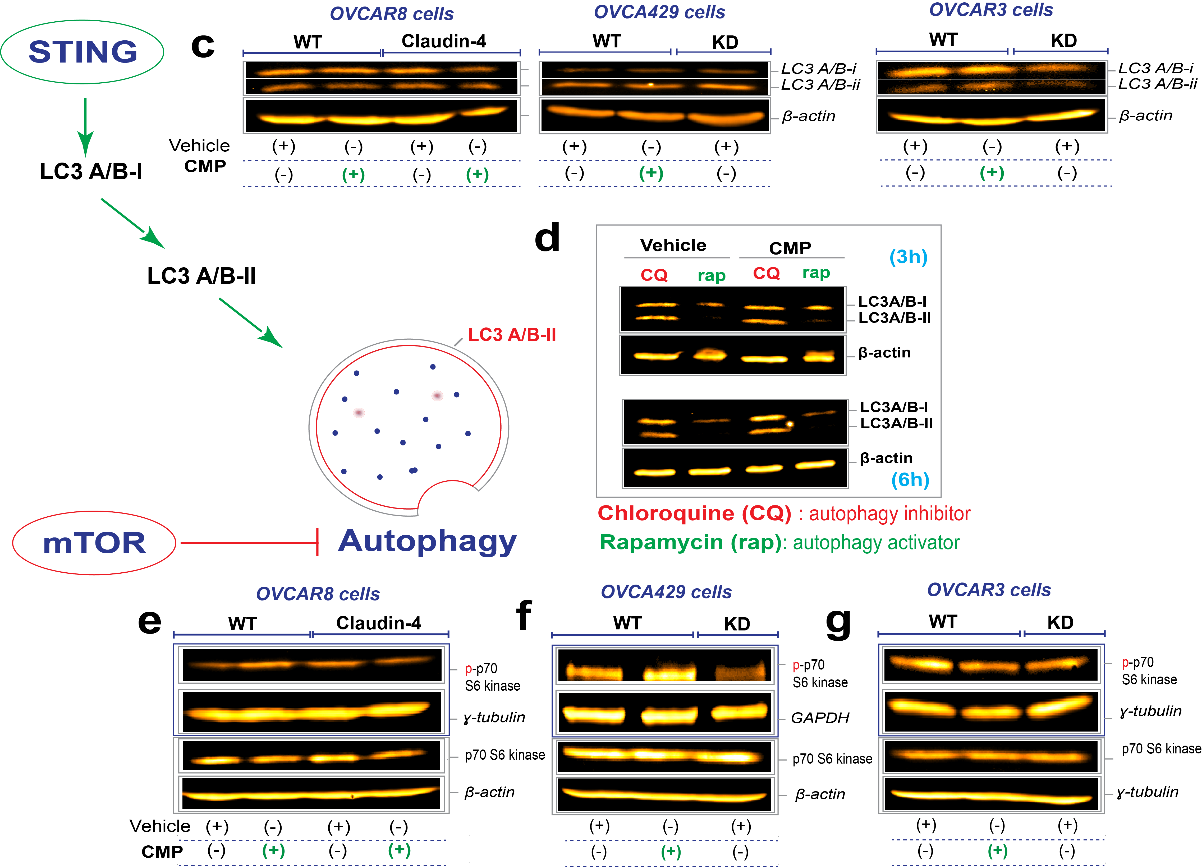


**Supplementary Figure 1. (a)** Verification of claudin-4 overexpression (top) in OVCAR8 cells (which do not express claudin-4) and its downregulation (knockdown using CRISPRi in OVCA429 and OVCAR3 cells) (bottom) by immunoblotting (IB). **(b)** On the left, a drawing highlighting the structure of claudin-4 in red and green colors, where two extracellular loops (large and small) can be observed. It also illustrates the feasible *cis* and *trans* interaction of this protein at the plasma membrane, a key characteristic that determines the function of claudin-4. *Cis* and *trans* interaction of claudin-4 with other proteins is also expected, thus potentially interfering with claudin-4’s interactions with other proteins and its function. ^1-4^ The drawing also highlights a conserved sequence of claudin-4 (DFYNP) located in the second (small) extracellular loop, as well as the sequence of the claudin mimic peptide (CMP; DFYNP which is in D-conformation and stabilized by Glycine at the flanking ends of the peptide). On the right, the drawing shows known and predicted phosphosites in claudin-4, including a highlighted predicted and uncharacterized phosphosite at Y148, located in the conserved sequence in the small extracellular loop of claudin-4. This prediction was generated using phosphonet server (<http://www.phosphonet.ca/>). (**c**) Left, illustration to highlight the induction and inhibition of autophagy via STING and mTOR, respectively; right, IB for LC3 A/B-i and ii (cell lysates from 48h). (**d**) IB for LC3 A/B-i and ii from cells treated with CQ (40µM) and rapamycin (8µM). (**e, f,** and **g**) IB for phospho (p)p70 S6 kinase (cell lysates from 48h).

**Supplementary References:**

1. Gehne, N., Lamik, A., Lehmann, M., Haseloff, R.F., Andjelkovic, A.V., and Blasig, I.E. (2017). Cross-over endocytosis of claudins is mediated by interactions via their extracellular loops. PloS one *12*, e0182106. 10.1371/journal.pone.0182106.

2. Piontek, J., Winkler, L., Wolburg, H., Muller, S.L., Zuleger, N., Piehl, C., Wiesner, B., Krause, G., and Blasig, I.E. (2008). Formation of tight junction: determinants of homophilic interaction between classic claudins. FASEB J *22*, 146-158. 10.1096/fj.07-8319com.

3. Baumgartner, H.K., Beeman, N., Hodges, R.S., and Neville, M.C. (2011). A D-peptide analog of the second extracellular loop of claudin-3 and -4 leads to mislocalized claudin and cellular apoptosis in mammary epithelial cells. Chem Biol Drug Des *77*, 124-136. 10.1111/j.1747-0285.2010.01061.x.

4. Hicks, D.A., Galimanis, C.E., Webb, P.G., Spillman, M.A., Behbakht, K., Neville, M.C., and Baumgartner, H.K. (2016). Claudin-4 activity in ovarian tumor cell apoptosis resistance and migration. BMC Cancer *16*, 788. 10.1186/s12885-016-2799-7.
