# Supplementary material for: Claudin-4 Modulates Autophagy via SLC1A5/LAT1 as a Mechanism to Regulate Micronuclei
Source: Cancer Res Commun. 2024 Jul 2;4(7):1625–42. doi: 10.1158/2767-9764.CRC-24-0240 (PMC11218812; doi:10.1158/2767-9764.CRC-24-0240)
Supplement: Supplementary Table 2 — Metabolomics OVCA429 and OVCAR3 [file crc-24-0240_supplementary_table_2_suppst2.docx]

Supplemental Table 2, Villagomez 2024

OVCA429 Cells

|  | **OVCA429 Cells** | | | | | |
| --- | --- | --- | --- | --- | --- | --- |
|  | **WT** | **WT** | **WT** | **CLDN4 KD** | **CLDN4 KD** | **CLDN4 KD** |
| **compound** | **DS2-041-001+** | **DS2-041-002+** | **DS2-041-003+** | **DS2-041-004+** | **DS2-041-005+** | **DS2-041-006+** |
| L-alanine | 3836680 | 2911700 | 4268960 | 7060834 | 3375735 | 9301574 |
| L-arginine | 13024717 | 13060240 | 12083693 | 4489164 | 12980396 | 15693904 |
| L-asparagine | 1091188 | 1189868 | 1546986 | 1805597 | 978435 | 2925022 |
| L-aspartate | 27705008 | 22444114 | 20861762 | 16564516 | 19594230 | 21992462 |
| L-cysteine | 517121 | 165798 | 180381 | 170193 | 276949 | 422138 |
| L-glutamate | 41640644 | 44715236 | 39299332 | 70300184 | 54265452 | 60016876 |
| L-glutamine | 2752012 | 2087131 | 4367734 | 4507660 | 1838473 | 3726551 |
| glycine | 1622663 | 1223771 | 2323126 | 3117828 | 1829426 | 4302728 |
| L-histidine | 763439 | 690256 | 452878 | 572509 | 801589 | 1230153 |
| L-leucine/isoluecine | 58084372 | 40181568 | 27154214 | 41015888 | 58833536 | 75435512 |
| L-lysine | 6048903 | 6056031 | 2741384 | 2517665 | 6635602 | 7498478 |
| L-methionine | 5244682 | 4053288 | 2605220 | 4017228 | 5498558 | 7006329 |
| L-phenylalanine | 7270882 | 5263252 | 2917667 | 4963687 | 7290500 | 8103961 |
| L-proline | 98163688 | 72648408 | 64563052 | 112303520 | 81788448 | 87694248 |
| L-serine | 719614 | 649633 | 1135343 | 1243791 | 660250 | 1633742 |
| L-threonine | 1740712 | 1436749 | 1082606 | 1437287 | 1456479 | 2298081 |
| L-tryptophan | 17436534 | 11833159 | 5156743 | 8631660 | 16267949 | 16146593 |
| L-tyrosine | 8630867 | 6568927 | 4301167 | 5591313 | 9190998 | 10994627 |
| L-valine | 17761794 | 17317668 | 14259947 | 25496914 | 21239010 | 20007452 |
| L-cystine | 53143 | 37311 | 56181 | 105229 | 63585 | 100062 |
| ATP | 3211414 | 3816406 | 6920683 | 4031877 | 3912582 | 10550260 |
| AMP | 25408694 | 22112904 | 6911888 | 24568432 | 26207550 | 17761586 |
| Adenosine | 1114620 | 2129304 | 589633 | 601938 | 1340003 | 419451 |
| GTP | 245981 | 525284 | 900217 | 632087 | 538694 | 1215379 |
| GDP | 1347732 | 1670711 | 1349505 | 2661063 | 2349459 | 3295988 |
| GMP | 2315377 | 1259795 | 1363695 | 3570578 | 2674024 | 4424266 |
| Guanine | 144509 | 110029 | 52178 | 66042 | 111398 | 137721 |
| CTP | 186842 | 335089 | 696677 | 529949 | 392738 | 923290 |
| CDP | 971491 | 976612 | 1071486 | 2417761 | 1420796 | 2479586 |
| Thymidine | 154308 | 47918 | 107374 | 200672 | 74351 | 107203 |
| UTP | 780059 | 1285370 | 3522528 | 1857300 | 1462935 | 2864352 |
| UDP | 3704991 | 4627321 | 5576245 | 8476809 | 5750111 | 7801912 |
| Uracil | 172988 | 228240 | 195167 | 266282 | 372785 | 66372 |
| IMP | 307061 | 251838 | 167769 | 81398 | 616586 | 922965 |
| Inosine | 4338279 | 2122250 | 1976287 | 3508944 | 4569400 | 9049265 |
| Hypoxanthine | 9131913 | 6752813 | 5043656 | 13089701 | 7433783 | 7483395 |
| Xanthine | 135686 | 58845 | 191749 | 35164 | 101598 | 73662 |
| Urate | 96953 | 87504 | 88912 | 125319 | 217038 | 211774 |
| Allantoate | 673233 | 555658 | 482960 | 588432 | 514638 | 648238 |
| 5-6-Dihydrothymine | 107466 | 96277 | 104111 | 153390 | 130958 | 151676 |
| 4-Pyridoxate | 622555 | 654973 | 416853 | 750287 | 425630 | 727460 |
| Nicotinamide | 4961851 | 4288000 | 2011213 | 2881020 | 4649492 | 2353979 |
| Adenylosuccinic acid | 469730 | 400945 | 49831 | 470688 | 495634 | 263061 |
| UDP-glucose | 6441051 | 5580046 | 3680319 | 10854029 | 7921652 | 11632888 |
| ADP-D-ribose | 456306 | 332352 | 1078979 | 158398 | 413010 | 431451 |
| NADP+ | 170887 | 65871 | 407281 | 420712 | 356653 | 772449 |
| NAD+ | 4979664 | 4328072 | 4524611 | 9598816 | 5518869 | 11151756 |
| NADH | 315479 | 169634 | 168078 | 440311 | 295034 | 566955 |
| Phosphate | 250973040 | 291776480 | 213077248 | 196011920 | 267628800 | 186044304 |
| Diphosphate | 39857532 | 64434724 | 33045818 | 34749920 | 57787460 | 27116072 |
| D-Glucose | 5801359 | 4095034 | 3495691 | 7574636 | 5963022 | 11096349 |
| D-Hexose-phosphate | 4712762 | 3624534 | 4869737 | 4692157 | 4726714 | 4470377 |
| D-Fructose 1-6-bisphosphate | 6725033 | 5935545 | 12260659 | 6638213 | 9185188 | 7495652 |
| D-Glyceraldehyde 3-phosphate/Glycerone phosphate | 2413525 | 1992706 | 5463006 | 1172463 | 1958998 | 1234813 |
| 1-3-Bisphosphoglycerate | 328189 | 2832043 | 901022 | 1728614 | 3882519 | 1354204 |
| 2/3-Phospho-D-glycerate | 12546725 | 9008897 | 13298148 | 2451530 | 9182740 | 4411613 |
| Phosphoenolpyruvate | 768219 | 770961 | 751037 | 256022 | 1093368 | 303191 |
| Pyruvate | 1019920 | 533096 | 1025032 | 1603104 | 855144 | 987601 |
| Lactate | 49414868 | 20629948 | 33774204 | 81411912 | 35378820 | 72237720 |
| Maltose/Sucrose | 1856934 | 809285 | 802213 | 868835 | 1132712 | 2014054 |
| Mannitol/Sorbitol/Glucitol/Iditol | 7364696 | 8005096 | 4303776 | 7071354 | 9004255 | 6663349 |
| Ribose/Ribulose/Arabinose/Xylose/Xylulose | 254632 | 167936 | 129842 | 313682 | 210471 | 389126 |
| D-Arabitol/Xylitol/Ribitol | 841597 | 625221 | 680933 | 1131979 | 1021518 | 1289344 |
| Citrate | 51633408 | 26294768 | 14293273 | 33579680 | 36667028 | 29936056 |
| 2-Oxoglutarate | 289073 | 173892 | 154441 | 613647 | 263680 | 218018 |
| 2-Oxoglutaramate | 113026 | 67917 | 409794 | 369140 | 124751 | 327124 |
| Succinate | 7531961 | 6451496 | 3840893 | 11195383 | 6000298 | 7655737 |
| Fumarate | 3901088 | 2126925 | 2191904 | 2547264 | 2445823 | 2253743 |
| Malate | 73374168 | 42597180 | 41534732 | 50263984 | 51886964 | 42192452 |
| Oxaloacetate | 71941 | 8203 | 22330 | 39750 | 73097 | 20798 |
| 2-Hydroxyglutarate | 1835173 | 1413609 | 1967556 | 7788420 | 1927257 | 1754740 |
| 6-Phospho-D-gluconate | 955768 | 954869 | 472608 | 871785 | 912786 | 503470 |
| Glutathione | 253644560 | 177614656 | 178931632 | 280994400 | 225361920 | 254900944 |
| Glutathione disulfide | 331961 | 342073 | 330108 | 330028 | 475286 | 929613 |
| 5-Oxoproline | 4487574 | 2930407 | 3025743 | 4426705 | 3531719 | 3049632 |
| S-Glutathionyl-L-cysteine | 230339 | 112901 | 270979 | 341789 | 274133 | 617946 |
| Cys-Gly | 1281593 | 1071031 | 969650 | 1066530 | 1236209 | 1368823 |
| Ascorbate | 295411 | 295089 | 205200 | 253035 | 290591 | 521997 |
| Dehydroascorbate | 998019 | 564865 | 261484 | 817316 | 769562 | 634707 |
| gamma-Glutamyl-Se-methylselenocysteine | 51684 | 39710 | 43480 | 39157 | 40917 | 51062 |
| gamma-Glutamyl-gamma-aminobutyrate | 131023 | 86187 | 147451 | 36010 | 308186 | 255574 |
| Cystathionine | 314714 | 199815 | 590149 | 855845 | 393722 | 626654 |
| Dimethylglycine | 596834 | 504147 | 394362 | 723169 | 470845 | 449693 |
| S-Adenosyl-L-methionine | 498269 | 224586 | 376902 | 542077 | 315481 | 268540 |
| Ornithine | 553443 | 288828 | 262841 | 630061 | 294014 | 645624 |
| Argininosuccinate | 306875 | 318170 | 215091 | 180114 | 403290 | 525132 |
| Putrescine | 879284 | 569667 | 786109 | 1381746 | 621718 | 465750 |
| Spermidine | 2415212 | 2754557 | 1052905 | 2522578 | 4738692 | 1684516 |
| N-Acetylneuraminate | 29480 | 86269 | 38999 | 0 | 49102 | 66101 |
| UDP-N-acetyl-D-glucosamine | 12284893 | 10776753 | 7588658 | 12264096 | 13837441 | 25048000 |
| CMP-N-acetylneuraminate | 158749 | 175362 | 75088 | 66673 | 169589 | 249994 |
| Phosphocreatine | 2353546 | 1736974 | 1422613 | 3245773 | 2605901 | 1794184 |
| Creatine | 28374030 | 16926120 | 20645592 | 31166528 | 20514608 | 25623874 |
| Creatinine | 383071 | 212557 | 220161 | 411201 | 233513 | 351023 |
| N-Acetyl-L-ornithine | 817710 | 335818 | 328996 | 308957 | 554920 | 473661 |
| trans-4-Hydroxy-L-proline | 2594341 | 2004946 | 3196873 | 6110767 | 2441991 | 3685928 |
| N-Succinyl-L-glutamate 5-semialdehyde | 97165 | 100409 | 105457 | 231139 | 151425 | 370603 |
| Pantothenate | 3767496 | 4772449 | 4119606 | 4213712 | 6524512 | 2256592 |
| Pantetheine | 49987 | 36639 | 5963 | 18347 | 42010 | 34186 |
| Taurine | 3934463 | 4261917 | 6132468 | 8334531 | 4010654 | 12075944 |
| Hypotaurine | 145600 | 143968 | 146888 | 208471 | 64887 | 340594 |
| Indole-3-acetate | 679969 | 1452212 | 1526611 | 148721 | 1857847 | 540602 |
| Kynurenine | 112726 | 573673 | 177240 | 33156 | 433928 | 187105 |
| Anthranilate | 191026 | 155030 | 85415 | 135971 | 137067 | 103654 |
| g-Oxalo-crotonate | 600564 | 622259 | 311760 | 436580 | 441550 | 334146 |
| 2-Aminomuconate | 1856756 | 1573855 | 1648812 | 2228847 | 2134874 | 2194422 |
| Ectoine | 26850 | 35479 | 13973 | 41096 | 17441 | 17232 |
| Glycerol 3-phosphate | 585587 | 1961037 | 3975877 | 316354 | 1709810 | 548098 |
| Choline | 72683232 | 96943680 | 65436212 | 25395296 | 107729280 | 45299476 |
| Acetylcholine | 10823667 | 7585224 | 6251352 | 10490003 | 8143236 | 8645163 |
| L-Carnitine | 33115048 | 17092814 | 15193999 | 30998142 | 18285914 | 27959718 |
| AC(2:0) | 25766848 | 19106318 | 11238959 | 21077018 | 35713116 | 23621112 |
| AC(3:0) | 7956460 | 5289084 | 3885983 | 2390305 | 6181532 | 3645348 |
| AC(4:0) | 41225476 | 21146326 | 18257766 | 59715948 | 43352996 | 50008000 |
| AC(4-OH) | 971106 | 702431 | 433575 | 814804 | 974160 | 558703 |
| AC(5:0) | 53226196 | 25649088 | 11634580 | 24519942 | 40110540 | 40891668 |
| AC(5:1) | 247282 | 267487 | 57581 | 18249 | 115861 | 19628 |
| AC(5-OH) | 733293 | 870255 | 424255 | 314338 | 1018368 | 516378 |
| AC(6:0) | 5705875 | 4624932 | 3121983 | 3862432 | 3355499 | 1924316 |
| AC(8:0) | 2387817 | 1632087 | 1073053 | 1108169 | 1256819 | 543264 |
| AC(10:0) | 708359 | 520829 | 418445 | 403851 | 554138 | 369648 |
| AC(10:1) | 143091 | 32695 | 70973 | 153007 | 69343 | 25064 |
| AC(12:0) | 3486279 | 4431302 | 3129556 | 3374686 | 5085152 | 3225757 |
| AC(12:1) | 944119 | 288053 | 230694 | 215790 | 432061 | 534850 |
| AC(14:0) | 30729446 | 82747512 | 68826256 | 47689836 | 74453128 | 39214320 |
| AC(14:1) | 4787829 | 2661643 | 2690061 | 1889231 | 3767722 | 7196835 |
| AC(16:0) | 27669504 | 30772406 | 10996456 | 18994862 | 29943216 | 27331664 |
| AC(16:1) | 16822408 | 10625075 | 11188861 | 10879441 | 15051373 | 39792420 |
| AC(18:0) | 1125638 | 5689620 | 1643437 | 856721 | 5804092 | 1386075 |
| AC(18:1) | 8931891 | 9385209 | 4913992 | 4566874 | 9982047 | 18188764 |
| AC(18:2) | 1229600 | 1635335 | 1037817 | 629151 | 1513505 | 3516220 |
| AC(20:4) | 808992 | 1321744 | 614055 | 471568 | 1110251 | 1484038 |
| FA(5:0) | 871110 | 803249 | 699927 | 772443 | 761642 | 838573 |
| FA(6:0) | 4231810 | 3223065 | 2794042 | 3682907 | 4345536 | 3220209 |
| FA(7:0) | 10712732 | 9647701 | 10919356 | 10921879 | 11022395 | 10362387 |
| FA(8:0) | 36765076 | 26558160 | 28072602 | 31015678 | 28271568 | 26799856 |
| FA(9:0) | 43111280 | 37481964 | 40655004 | 48262688 | 51859184 | 49073216 |
| FA(10:0) | 70226776 | 53853740 | 61483724 | 68515928 | 73212456 | 64703284 |
| FA(12:0) | 74363760 | 64188500 | 72622408 | 76011976 | 73198728 | 68690912 |
| FA(14:0) | 98227968 | 155339904 | 237420368 | 377003680 | 230156672 | 85462936 |
| FA(16:0) | 232918080 | 406362880 | 236915328 | 200198192 | 268270192 | 270783520 |
| FA(18:0) | 148091376 | 322914464 | 142383488 | 115981160 | 186971664 | 201888896 |
| FA(14:1) | 4440167 | 4994516 | 2931595 | 3300089 | 5432535 | 4328156 |
| FA(16:1) | 38173344 | 26128594 | 13686180 | 25551782 | 38078720 | 33104154 |
| FA(18:1) | 91070248 | 116030360 | 51507732 | 60290700 | 114849248 | 91173480 |
| FA(18:2) | 12368948 | 23083722 | 6780613 | 8215804 | 22947194 | 11007657 |
| FA(18:3) | 954071 | 1527649 | 394769 | 468440 | 1033405 | 497342 |
| FA(20:4) | 81940568 | 115696776 | 53043188 | 58141204 | 109841192 | 49693856 |
| FA(20:5) | 3193480 | 12904584 | 1671734 | 1190220 | 6013440 | 1559376 |
| FA(22:6) | 10096375 | 23781666 | 7802019 | 5745438 | 22655616 | 8222538 |
| FA(20:3) | 5713453 | 11124769 | 3817229 | 5325354 | 10740479 | 5708330 |
| FA(22:5) | 9634282 | 20099448 | 5813894 | 5445104 | 18233794 | 6484854 |
| Sphingosine | 158091792 | 96784104 | 56588116 | 46858268 | 124405624 | 83923968 |
| Sphinganine 1-phosphate | 202334 | 401646 | 213498 | 191439 | 272469 | 346553 |
| 3-Phospho-D-glyceroyl phosphate | 328189 | 2832043 | 901022 | 1728614 | 3882519 | 1354204 |

OVCA429 Supernatant

|  | **OVCA429 Supernatant** | | | | | |
| --- | --- | --- | --- | --- | --- | --- |
|  | **WT** | **WT** | **WT** | **CLDN4 KD** | **CLDN4 KD** | **CLDN4 KD** |
| **compound** | **DS2-041-007+** | **DS2-041-008+** | **DS2-041-009+** | **DS2-041-010+** | **DS2-041-011+** | **DS2-041-012+** |
| L-alanine | 7027116 | 4601503 | 3934774 | 7243948 | 5437066 | 7202847 |
| L-arginine | 61328400 | 55950652 | 62189984 | 59663804 | 61324380 | 60843984 |
| L-asparagine | 2132336 | 2285425 | 1945027 | 2783411 | 2460028 | 2537542 |
| L-aspartate | 7257458 | 7858400 | 11123008 | 10152004 | 7612145 | 8699877 |
| L-cysteine | 462217 | 520626 | 336233 | 466261 | 550828 | 416380 |
| L-glutamate | 17986354 | 14804936 | 29011360 | 23495024 | 17548286 | 20400534 |
| L-glutamine | 18466000 | 25019944 | 16219400 | 14480999 | 18452274 | 18920744 |
| glycine | 835571 | 795243 | 860580 | 919655 | 947167 | 917664 |
| L-histidine | 2267193 | 2569508 | 2484156 | 2117990 | 2257652 | 2185729 |
| L-leucine/isoluecine | 128102048 | 132712856 | 171475888 | 138210448 | 151847856 | 126242248 |
| L-lysine | 13045371 | 17079122 | 17893862 | 13202731 | 18940602 | 12772800 |
| L-methionine | 17725176 | 20785142 | 22311730 | 17149280 | 19724822 | 18737558 |
| L-phenylalanine | 12081117 | 15906084 | 17960296 | 11719921 | 14682480 | 14232659 |
| L-proline | 62333972 | 68482760 | 65505396 | 54073312 | 65098196 | 60077108 |
| L-serine | 1126981 | 1387305 | 1195134 | 1325893 | 1131868 | 1555541 |
| L-threonine | 3460391 | 3422481 | 3513000 | 3143711 | 3589559 | 3791032 |
| L-tryptophan | 41492808 | 53057328 | 55116436 | 34353000 | 39256692 | 45816844 |
| L-tyrosine | 24186464 | 26975234 | 30414662 | 23094664 | 27127264 | 25857624 |
| L-valine | 49635596 | 54088324 | 63801812 | 47582156 | 55748636 | 56515412 |
| L-cystine | 9096221 | 11104880 | 9004409 | 8674011 | 10090913 | 10372275 |
| ADP | 0 | 2271 | 4485 | 2903 | 4760 | 4523 |
| AMP | 206953 | 110523 | 76769 | 121327 | 153792 | 227303 |
| GMP | 197999 | 108388 | 52576 | 174897 | 41432 | 136869 |
| Guanine | 311547 | 404174 | 484078 | 309318 | 357628 | 349503 |
| dCMP | 128242 | 148863 | 158452 | 138291 | 153175 | 129606 |
| Cytidine | 250502 | 188011 | 0 | 112626 | 158267 | 146996 |
| Thymidine | 89085 | 50965 | 91103 | 50863 | 25141 | 66809 |
| UDP | 6287 | 11719 | 7236 | 13877 | 8654 | 6395 |
| Uracil | 59819 | 121752 | 33651 | 99021 | 42556 | 64454 |
| IDP | 714811 | 1477720 | 291642 | 147970 | 1006893 | 470561 |
| Hypoxanthine | 18006130 | 18273742 | 21814668 | 20177898 | 18226254 | 18099682 |
| Xanthine | 244193 | 434460 | 424050 | 325925 | 377154 | 300514 |
| Urate | 822103 | 635141 | 429392 | 214853 | 557974 | 424144 |
| (S)(+)-Allantoin | 715441 | 701551 | 960682 | 805096 | 784696 | 612118 |
| Allantoate | 387477 | 277321 | 616344 | 462604 | 334455 | 484640 |
| 3',5'-Cyclic IMP | 2506914 | 5106953 | 930183 | 476050 | 3499306 | 3136270 |
| 5-6-Dihydrothymine | 950962 | 1042113 | 789530 | 834399 | 912008 | 1043396 |
| Pyridoxal | 542127 | 334946 | 263260 | 442524 | 474458 | 449135 |
| 4-Pyridoxate | 292287 | 234006 | 207521 | 120613 | 210261 | 145240 |
| Nicotinamide | 1651179 | 1799444 | 2197898 | 1823395 | 1855723 | 1708943 |
| Nicotinate ribonucleotide | 311912 | 374764 | 396227 | 224076 | 348364 | 361259 |
| Adenylosuccinic acid | 78140 | 115618 | 39633 | 126861 | 98918 | 104443 |
| ADP-D-ribose | 454389 | 595786 | 659451 | 483922 | 570385 | 661984 |
| Phosphate | 181540352 | 205111248 | 205388752 | 193273072 | 223749200 | 235892224 |
| Diphosphate | 29153626 | 33847348 | 22490000 | 31960424 | 29650472 | 32551778 |
| D-Glucose | 41435888 | 45894644 | 51546832 | 39942336 | 41068124 | 39296084 |
| D-Hexose-phosphate | 200921 | 135160 | 215301 | 91210 | 150175 | 231120 |
| 1-3-Bisphosphoglycerate | 1328 | 3001 | 0 | 8056 | 1643 | 1569 |
| 2/3-Phospho-D-glycerate | 64633 | 25666 | 41384 | 6750 | 34398 | 36611 |
| Pyruvate | 12651067 | 11571916 | 10023069 | 12754148 | 9800806 | 8970425 |
| Lactate | 353792288 | 283940576 | 253697744 | 392429536 | 317400704 | 303816704 |
| Maltose/Sucrose | 3874974 | 4101003 | 2882899 | 3231543 | 4150218 | 4605912 |
| Maltotriose | 0 | 30121 | 79777 | 41945 | 39183 | 33508 |
| Mannitol/Sorbitol/Glucitol/Iditol | 14423929 | 12489461 | 16298701 | 19296698 | 14421065 | 15476048 |
| Ribose/Ribulose/Arabinose/Xylose/Xylulose | 1577651 | 1777174 | 1871003 | 1441228 | 1522217 | 1339519 |
| D-Rhamnose | 162697 | 74265 | 33728 | 158733 | 122469 | 202203 |
| D-Arabitol/Xylitol/Ribitol | 691581 | 505938 | 573961 | 605863 | 574395 | 474689 |
| Citrate | 27086008 | 25525446 | 39000100 | 26732290 | 26653856 | 26102234 |
| 2-Oxoglutarate | 2681568 | 1902003 | 3552079 | 2046021 | 2189330 | 1551522 |
| 2-Oxoglutaramate | 663032 | 497910 | 184618 | 601420 | 479772 | 531072 |
| Succinate | 19826138 | 22726670 | 24946786 | 20943694 | 18162658 | 19931970 |
| Fumarate | 741634 | 578320 | 799385 | 550598 | 638340 | 553974 |
| Malate | 13077915 | 11107195 | 15340043 | 10835898 | 12275533 | 8924764 |
| Oxaloacetate | 48602 | 9554 | 48908 | 21370 | 13362 | 70159 |
| 2-Hydroxyglutarate | 975590 | 825236 | 1278326 | 1031431 | 1270224 | 1095452 |
| 6-Phospho-D-gluconate | 23807 | 57152 | 39963 | 32433 | 34497 | 17128 |
| Glutathione | 106576 | 69808 | 30597 | 56387 | 76301 | 31164 |
| 5-Oxoproline | 42295376 | 46190132 | 54389428 | 53171716 | 47080148 | 40747804 |
| S-Glutathionyl-L-cysteine | 696556 | 643749 | 611263 | 511115 | 698047 | 613806 |
| Ascorbate | 457193 | 630480 | 667769 | 302564 | 487487 | 582898 |
| Dehydroascorbate | 943390 | 828202 | 1121602 | 786177 | 920658 | 852560 |
| gamma-L-Glutamyl-D-alanine | 146044 | 78360 | 98027 | 50836 | 143116 | 102407 |
| Dimethylglycine | 172005 | 178078 | 321430 | 273580 | 158333 | 233907 |
| Phosphoserine | 283390 | 135631 | 125432 | 197913 | 128693 | 193864 |
| S-Adenosyl-L-homocysteine | 0 | 0 | 0 | 0 | 30144 | 0 |
| Folate | 1243802 | 1363591 | 1556542 | 1303919 | 1152701 | 1107979 |
| 10-Formyltetrahydrofolate | 402125 | 346624 | 108803 | 345936 | 354033 | 463684 |
| Ornithine | 3048444 | 2649914 | 5514814 | 5736510 | 3160048 | 3572072 |
| L-Citrulline | 572887 | 487731 | 404194 | 549345 | 504878 | 469380 |
| Argininosuccinate | 39049 | 104689 | 78080 | 108416 | 64943 | 116094 |
| Putrescine | 50293 | 31486 | 78592 | 29567 | 26276 | 0 |
| Spermidine | 103503 | 241310 | 193843 | 120584 | 178761 | 117869 |
| N-Acetylneuraminate | 236951 | 259914 | 293101 | 245256 | 241356 | 298262 |
| N-Glycoloyl-neuraminate | 559204 | 695269 | 838813 | 727648 | 575688 | 735629 |
| Carnosine | 177048 | 175398 | 159812 | 149352 | 202113 | 132504 |
| Creatine | 26975842 | 29660874 | 26970336 | 25122642 | 26055994 | 28277240 |
| Creatinine | 19377038 | 16914478 | 15380116 | 16946616 | 17834026 | 16867562 |
| N-Acetyl-L-ornithine | 628380 | 526592 | 700261 | 607046 | 543492 | 538356 |
| trans-4-Hydroxy-L-proline | 8362474 | 7216242 | 7198024 | 8232554 | 7002244 | 7679768 |
| N-Succinyl-L-glutamate 5-semialdehyde | 656519 | 401780 | 541503 | 446783 | 638828 | 616321 |
| Pantothenate | 0 | 17922 | 15230 | 3572 | 2798 | 0 |
| Pantetheine | 717093 | 863458 | 495137 | 431123 | 801163 | 887834 |
| Taurine | 165993 | 178078 | 132214 | 151630 | 129133 | 145506 |
| L-Methionine S-oxide | 242222 | 271342 | 335692 | 354435 | 278960 | 320841 |
| Indole-3-acetate | 12490824 | 68425704 | 60498816 | 9529749 | 59632436 | 39341924 |
| Kynurenine | 142238 | 125122 | 121076 | 87930 | 118015 | 95717 |
| Kynurenic acid | 211645 | 265790 | 363524 | 263297 | 277416 | 280668 |
| Anthranilate | 141061 | 156887 | 163220 | 102415 | 123931 | 117109 |
| g-Oxalo-crotonate | 1168341 | 1354149 | 1527571 | 1242156 | 1232211 | 1352998 |
| 2-Aminomuconate | 853378 | 639452 | 685213 | 631217 | 761244 | 877335 |
| Ectoine | 155658 | 127611 | 139619 | 96832 | 147536 | 110789 |
| Phosphoethanolamine | 27437 | 70276 | 50011 | 60784 | 29419 | 41797 |
| CDP-choline | 33082 | 50812 | 45850 | 52808 | 14785 | 0 |
| Choline | 56999476 | 67087372 | 74549048 | 68608472 | 69665432 | 65219436 |
| Acetylcholine | 1110590 | 1141978 | 1597749 | 1080152 | 1145023 | 1086407 |
| L-Carnitine | 3290386 | 3224831 | 3548528 | 3545024 | 3506807 | 3296346 |
| AC(2:0) | 5908000 | 5680814 | 7921538 | 6181013 | 6245327 | 5719730 |
| AC(3:0) | 2260720 | 2311867 | 3317274 | 2462501 | 2328158 | 2035553 |
| AC(4:0) | 2951959 | 2316858 | 2589150 | 2200452 | 2660322 | 2126866 |
| AC(4-OH) | 116096 | 124031 | 151508 | 132532 | 115743 | 148815 |
| AC(4-DC) | 173502 | 231946 | 249670 | 212491 | 218236 | 166479 |
| AC(5:0) | 1359311 | 1557787 | 1729331 | 1214537 | 1408991 | 1314166 |
| AC(5:1) | 32260 | 32368 | 60539 | 63275 | 31264 | 51496 |
| AC(5-OH) | 75943 | 82917 | 86772 | 101983 | 109735 | 55840 |
| AC(8:0) | 692744 | 670831 | 536030 | 498817 | 266759 | 238563 |
| AC(10:0) | 435011 | 540744 | 297313 | 299112 | 213321 | 182247 |
| AC(10:1) | 42307 | 56830 | 33670 | 67705 | 17006 | 28028 |
| AC(12:0) | 188460 | 288590 | 252542 | 160048 | 125446 | 62079 |
| AC(12:1) | 138916 | 122599 | 155500 | 124788 | 72719 | 49390 |
| AC(14:0) | 201033 | 278751 | 275498 | 139216 | 111521 | 76921 |
| AC(14:1) | 160020 | 262161 | 212477 | 169066 | 57966 | 26481 |
| AC(16:0) | 146979 | 134655 | 134414 | 76677 | 314740 | 103724 |
| AC(16:1) | 181596 | 167978 | 167921 | 166337 | 68463 | 98956 |
| AC(18:0) | 1625212 | 1406259 | 1347206 | 784605 | 4979948 | 1712681 |
| AC(18:1) | 48772 | 76668 | 89147 | 43761 | 54389 | 36057 |
| FA(5:0) | 978054 | 991812 | 1032242 | 1002984 | 1015967 | 1109572 |
| FA(6:0) | 5439427 | 5480816 | 5863652 | 5163871 | 5480736 | 5573382 |
| FA(7:0) | 13144151 | 12977405 | 12463451 | 12763269 | 11925819 | 12214627 |
| FA(8:0) | 37631460 | 36129428 | 37485756 | 37438792 | 35423684 | 33625928 |
| FA(9:0) | 40240092 | 49793652 | 42774204 | 45751556 | 51078960 | 47081536 |
| FA(10:0) | 75898744 | 71702040 | 73333008 | 74422560 | 72898216 | 73235232 |
| FA(12:0) | 72375896 | 80406216 | 77599352 | 72824816 | 74842464 | 78619272 |
| FA(14:0) | 109706944 | 125922464 | 120146920 | 105563680 | 113164064 | 134223760 |
| FA(16:0) | 285828672 | 334601856 | 310787456 | 258034688 | 243767600 | 181723520 |
| FA(18:0) | 160257488 | 274696800 | 185914496 | 143954864 | 154948816 | 59784948 |
| FA(14:1) | 2247635 | 2389304 | 2650769 | 2379662 | 2203531 | 2295137 |
| FA(16:1) | 7748480 | 9864921 | 8782813 | 5827990 | 6959440 | 9767428 |
| FA(18:1) | 26054974 | 40377460 | 30937096 | 20180648 | 25141418 | 20905792 |
| FA(18:2) | 3243382 | 6031683 | 4278333 | 2603766 | 3500061 | 3899268 |
| FA(18:3) | 132466 | 311703 | 224633 | 146228 | 132495 | 159709 |
| FA(20:4) | 636727 | 3302731 | 2952942 | 479344 | 1206248 | 2594626 |
| FA(20:5) | 172376 | 402279 | 368150 | 237222 | 244414 | 229337 |
| FA(22:6) | 965299 | 3642781 | 3033388 | 849553 | 1527637 | 1922126 |
| FA(20:3) | 167854 | 513825 | 434618 | 96904 | 224695 | 573774 |
| FA(22:5) | 137304 | 544125 | 579385 | 104658 | 260854 | 393104 |
| Sphingosine | 160250 | 303961 | 166915 | 105674 | 92733 | 244321 |
| Sphingosine 1-phosphate | 15151 | 43338 | 0 | 13843 | 33209 | 11133 |
| Sphinganine 1-phosphate | 280131 | 417607 | 250280 | 191279 | 315844 | 91501 |
| 3-Phospho-D-glyceroyl phosphate | 1328 | 3001 | 0 | 8056 | 1643 | 1569 |

OVCAR3 Cells

|  | **OVCAR3 Cells** | | | | | |
| --- | --- | --- | --- | --- | --- | --- |
|  | **WT** | **WT** | **WT** | **CLDN4 KD** | **CLDN4 KD** | **CLDN4 KD** |
| **compound** | **DS2-026-001+** | **DS2-026-002+** | **DS2-026-003+** | **DS2-026-004+** | **DS2-026-005+** | **DS2-026-006+** |
| L-alanine | 1692345.62 | 1429751 | 2073386 | 1413125.5 | 1527271.38 | 2406127.5 |
| L-arginine | 5773253.5 | 4132678.75 | 4244693.5 | 5863876 | 6532252 | 5668031.5 |
| L-asparagine | 423264.53 | 726156.19 | 785719.56 | 829035.94 | 1076384.12 | 972101.94 |
| L-aspartate | 31361432 | 24396816 | 20560894 | 29137394 | 33890028 | 20054506 |
| L-cysteine | 263486.5 | 452230.41 | 468684 | 381188.5 | 503878 | 608412 |
| L-glutamate | 103060904 | 83469240 | 119252584 | 115229464 | 81223168 | 112420696 |
| L-glutamine | 1838126.62 | 4372680.5 | 2384053.75 | 4196525.5 | 6102878.5 | 4031977.75 |
| glycine | 2277713.25 | 531890.56 | 3078940 | 2951712.25 | 1117387.38 | 2421363.75 |
| L-histidine | 430429.41 | 685944.19 | 622598.31 | 505262.34 | 640247.56 | 715242.44 |
| L-leucine/isoluecine | 30901750 | 48701708 | 47374752 | 38911724 | 38205884 | 50919828 |
| L-lysine | 2506980 | 2396678.75 | 2929619.75 | 2900538.75 | 2472459 | 2968027.25 |
| L-methionine | 3310830 | 5129018.5 | 4492252.5 | 3993480.75 | 3980265.25 | 5940925.5 |
| L-phenylalanine | 5023281.5 | 5896917.5 | 6012796 | 5796366.5 | 6201988.5 | 6007673.5 |
| L-proline | 76626552 | 77133000 | 86255392 | 77960072 | 70557392 | 92842152 |
| L-serine | 1071667.12 | 342851.5 | 835503.19 | 1221679.88 | 561752.12 | 597104.69 |
| L-threonine | 745411.69 | 797550.19 | 1041433.56 | 933444.06 | 705867.31 | 1177801 |
| L-tryptophan | 11384235 | 12140300 | 9261055 | 10952701 | 12190137 | 9881409 |
| L-tyrosine | 4264966 | 5273907.5 | 6965192 | 4551776 | 5429781.5 | 6289216 |
| L-valine | 26775184 | 36984844 | 24967498 | 34332164 | 34649964 | 25795222 |
| L-cystine | 33249.55 | 17282.62 | 26204.36 | 46112.63 | 6929.31 | 17459.65 |
| ATP | 26468302 | 23174958 | 41086436 | 63167012 | 49845116 | 62394400 |
| AMP | 7333158.5 | 10265231 | 3992927.75 | 1104615.38 | 268519.41 | 1054353.38 |
| dAMP | 995469.31 | 1010358.81 | 1013311.5 | 847016 | 696424 | 389325.47 |
| Adenosine | 259557.25 | 289975.84 | 259313.8 | 248993.52 | 244518.89 | 243033.75 |
| Adenine | 528170.19 | 895543.69 | 843720.44 | 383979.66 | 146025.88 | 402989.75 |
| GTP | 2379691.5 | 1835515.38 | 3148610.75 | 3726868.75 | 3712750 | 4296346.5 |
| GDP | 3503336.25 | 3015192 | 3246866.75 | 2455435.75 | 2545402 | 3000372.25 |
| Guanine | 122449.79 | 155918.58 | 171652.45 | 144024.45 | 128726.23 | 182330.42 |
| CTP | 3191872.25 | 2873070.75 | 5231872.5 | 6923533.5 | 4660532.5 | 5916133.5 |
| CDP | 4511999 | 4048854 | 5112190 | 4782028 | 2729798 | 4264613.5 |
| CMP | 896894.94 | 422567.34 | 834673.56 | 229196.92 | 7314.86 | 225383.75 |
| dCMP | 20180.4 | 36958.11 | 23760.41 | 24462.55 | 26724.07 | 21287.92 |
| Cytidine | 1621615.62 | 925455.44 | 1684058.62 | 2221255.75 | 2578755.5 | 2293506.25 |
| Thymidine | 141241.86 | 261105.75 | 244269.52 | 237054.14 | 258470.19 | 342937.62 |
| UTP | 12473371 | 10305654 | 17006374 | 24770146 | 16086316 | 18068050 |
| UDP | 20749772 | 19869922 | 24812778 | 25250190 | 16749347 | 23021464 |
| UMP | 3562107.75 | 2612949.25 | 2014621.38 | 758765.19 | 115063.06 | 478514.91 |
| Uracil | 295919.84 | 231260.31 | 363887.78 | 367590 | 390130.09 | 360043.16 |
| ITP | 183274.55 | 94346.12 | 46186.32 | 146467.78 | 0 | 0 |
| IDP | 123689.96 | 45890.32 | 120111.71 | 117949.88 | 46278.05 | 155192.7 |
| IMP | 765114.69 | 358282.88 | 343848.91 | 292390.91 | 91553.77 | 420666.59 |
| Inosine | 1503954.5 | 1533539.12 | 1469816.38 | 1535228.5 | 1247002.88 | 986920.44 |
| Hypoxanthine | 13801951 | 13559588 | 18257190 | 13723952 | 9191450 | 15292663 |
| Xanthine | 57211.18 | 57040 | 63940.51 | 42755.82 | 34506.21 | 70582.34 |
| 5-Hydroxyisourate | 87505.02 | 407062.72 | 186531.64 | 293296.09 | 389539.03 | 272113.16 |
| Allantoate | 79071.32 | 128042.59 | 258227.69 | 116135.53 | 46496.96 | 75565.34 |
| 5'-Phosphoribosyl-N-formylglycinamide | 1508434.88 | 6246687.5 | 1400161.88 | 2933561 | 3999054.25 | 1837855.38 |
| 5-6-Dihydrothymine | 391460.25 | 397039.59 | 437876.91 | 315502.19 | 266931.41 | 369656.91 |
| 4-Pyridoxate | 552501.5 | 773330.5 | 764349.06 | 558774.94 | 380907.75 | 650397.31 |
| Nicotinamide | 3740845.25 | 2902608.25 | 2953044.75 | 4925468.5 | 3328056.75 | 4996972.5 |
| UDP-glucose | 10125129 | 7911651.5 | 13087424 | 10485420 | 4945857.5 | 7098716 |
| ADP-D-ribose | 172851.73 | 431974.88 | 296562.59 | 254825.12 | 432789.5 | 530795.62 |
| NADP+ | 318573.16 | 141830.19 | 476376.41 | 309476.91 | 430837.16 | 311626.25 |
| NAD+ | 10125337 | 10380628 | 12230427 | 12832731 | 10713655 | 12922776 |
| NADH | 480058.75 | 451374.5 | 576962.62 | 643216.44 | 590503.69 | 683849.31 |
| Phosphate | 197647424 | 186887872 | 173529024 | 155251456 | 179436672 | 121756728 |
| Diphosphate | 29929578 | 31849722 | 26384992 | 23597280 | 20461098 | 20837738 |
| D-Glucose | 14606021 | 15700512 | 18344078 | 9316859 | 9903673 | 11763148 |
| D-Hexose-phosphate | 3017993 | 5721308.5 | 4242302 | 2331814 | 4269972 | 2827572.25 |
| D-Fructose 1-6-bisphosphate | 3042972.25 | 8486565 | 8106250 | 3325725.25 | 8329394 | 4275693.5 |
| D-Glyceraldehyde 3-phosphate/Glycerone phosphate | 568784 | 1190530.12 | 1344033 | 445462.84 | 2172414.25 | 821629.81 |
| 1-3-Bisphosphoglycerate | 426856.38 | 197669.7 | 545541.19 | 1085011.12 | 851432.31 | 718968 |
| 2/3-Phospho-D-glycerate | 859189.75 | 2115553 | 1370349.62 | 1001555 | 3595747.25 | 1482318.88 |
| Phosphoenolpyruvate | 87877 | 134808.62 | 133957.83 | 144911.8 | 365472.16 | 172671.55 |
| Pyruvate | 973692.5 | 1537692.62 | 1528799.12 | 1239047.25 | 1681512.12 | 1773172.12 |
| Lactate | 25973934 | 44628428 | 47138772 | 18795128 | 38647652 | 56111748 |
| Maltose/Sucrose | 1814103.12 | 1721254 | 1370554.88 | 1978567.38 | 1501347.5 | 1641765 |
| Mannitol/Sorbitol/Glucitol/Iditol | 6540986.5 | 5047972.5 | 7049256 | 10468108 | 5458601 | 6144917.5 |
| Ribose/Ribulose/Arabinose/Xylose/Xylulose | 403442.84 | 273601.84 | 444094.66 | 464441.09 | 388365.59 | 505146.22 |
| D-Arabitol/Xylitol/Ribitol | 966703.06 | 786253.5 | 882558.31 | 849776.19 | 517657.75 | 1071043.38 |
| Citrate | 144296224 | 121969984 | 126847016 | 169652080 | 107379456 | 148955568 |
| 2-Oxoglutarate | 1652525 | 1188430.62 | 2125274.5 | 2987530.25 | 1266031.88 | 2520980.25 |
| Succinate | 6830078.5 | 9935460 | 8701122 | 5926654 | 3034822 | 5758227.5 |
| Fumarate | 7575954 | 6520257.5 | 6707566.5 | 7023646.5 | 6409071.5 | 6654665.5 |
| Malate | 112796736 | 104746368 | 105764832 | 109489696 | 94432512 | 128952440 |
| Oxaloacetate | 145890.62 | 150876.48 | 129053.22 | 168407.86 | 161043.08 | 125252.95 |
| 2-Hydroxyglutarate | 8320580 | 5682692 | 6724250 | 7382804 | 4571742.5 | 7448536 |
| 6-Phospho-D-gluconate | 284583.44 | 657334.56 | 688460.25 | 314878.53 | 458441 | 468895.66 |
| Glutathione | 315424224 | 280705248 | 357272704 | 263693584 | 225554688 | 289080608 |
| Glutathione disulfide | 443002.84 | 363451.62 | 199408 | 272206.59 | 259187.92 | 187994.44 |
| 5-Oxoproline | 5782879.5 | 9532271 | 8536325 | 8335325.5 | 8235649.5 | 9260365 |
| Cys-Gly | 2571336.25 | 2414191.75 | 2869637 | 2217272.75 | 1896459 | 2200955.25 |
| Ascorbate | 991234.06 | 1231290.62 | 1427166.5 | 781744.81 | 940468 | 1460790.62 |
| Dehydroascorbate | 3136021.75 | 2948164.75 | 2469621 | 3749135.25 | 2285210.25 | 3388972 |
| gamma-L-Glutamyl-L-cysteine | 734479.44 | 624716 | 486534.16 | 604656.56 | 649857.94 | 567530.56 |
| gamma-Glutamyl-Se-methylselenocysteine | 29108.38 | 72396.22 | 50050.94 | 26124.77 | 22216.51 | 29466.68 |
| gamma-L-Glutamyl-D-alanine | 209321.38 | 204136.89 | 182443.19 | 230981.67 | 129197.75 | 175117.33 |
| Dimethylglycine | 1177237.25 | 1285659.75 | 1314319.88 | 1006566 | 1065117.12 | 1217333.12 |
| S-Adenosyl-L-methionine | 600344.44 | 890391.06 | 778929.69 | 951740.94 | 1214480.75 | 1149718.88 |
| Ornithine | 341972.88 | 328275.56 | 559059.31 | 283450.28 | 245431.55 | 343034 |
| Argininosuccinate | 447231.59 | 346687.22 | 155682.73 | 379559.84 | 334350.34 | 490973.47 |
| Spermidine | 3293712 | 5209111.5 | 4429343.5 | 3668328 | 4288071.5 | 4763164.5 |
| Spermine | 4904443 | 4468635.5 | 5853172.5 | 9963089 | 5276882.5 | 7147062.5 |
| N-Acetylneuraminate | 453454.09 | 274251.53 | 509294.34 | 395036.97 | 338554.47 | 408008.75 |
| N-Glycoloyl-neuraminate | 274540.22 | 223067.14 | 330978.03 | 222186.67 | 337164.5 | 317187.72 |
| UDP-N-acetyl-D-glucosamine | 43831388 | 25167816 | 34051012 | 28783402 | 28748774 | 44781780 |
| CMP-N-acetylneuraminate | 1237329.88 | 1171090.38 | 1309296.38 | 1388110.62 | 920170.31 | 1208193.12 |
| Carnosine | 106340.8 | 127155.16 | 235936.23 | 95625 | 256594.56 | 259478.02 |
| Phosphocreatine | 3152787.75 | 3309746 | 3754455.25 | 5342408.5 | 4256488 | 3956095.75 |
| Creatine | 128125112 | 120369856 | 124715648 | 96205896 | 69248448 | 109164288 |
| Creatinine | 628828.5 | 616135.44 | 706924.19 | 678553.62 | 677541.69 | 672997.81 |
| N-Acetyl-L-ornithine | 280468.03 | 433516.22 | 315828.97 | 199885.95 | 284056.41 | 214649.25 |
| trans-4-Hydroxy-L-proline | 1499389.62 | 2717653.75 | 2998849.25 | 2429443.5 | 3473012.75 | 4072476.75 |
| Pantothenate | 955253.56 | 1603454.5 | 1134655.88 | 509666.97 | 992441.5 | 799322.06 |
| Pantetheine | 30544.34 | 29430.1 | 32801.24 | 45925.8 | 19816.57 | 24841.27 |
| Taurine | 2399040.75 | 1765676.5 | 1944490.38 | 1130849.88 | 756547.69 | 878583.19 |
| 5-Hydroxyindoleacetate | 266231.12 | 82291.52 | 227968.3 | 566712.44 | 64192.91 | 252193.73 |
| Indole-3-acetate | 1305383.12 | 1427753.62 | 701620.81 | 1259708.12 | 916630.69 | 488656.12 |
| Indolepyruvate | 97637.65 | 52478.74 | 37296.6 | 94557.38 | 122009.96 | 130834.71 |
| Quinolinic acid | 82136.85 | 34715.51 | 62044.03 | 38973.93 | 66714.38 | 27636.22 |
| Picolinic acid | 809166.81 | 635359.75 | 994782.75 | 1062643.88 | 994108.81 | 1031189 |
| g-Oxalo-crotonate | 552700.69 | 1399470.62 | 993415.19 | 497770.5 | 625229.69 | 753136.75 |
| 2-Aminomuconate | 1058950.75 | 838022.19 | 1210654.88 | 741528.75 | 699469.25 | 1366144.38 |
| Glycerol 3-phosphate | 613920.25 | 777866 | 1502307 | 691610.94 | 1329947.38 | 1059356.38 |
| Phosphoethanolamine | 862495.06 | 172608.91 | 1447454.62 | 707169 | 401617.16 | 809131.5 |
| N-Methylethanolamine phosphate | 120329.05 | 119316.77 | 238622.61 | 409985.38 | 347950.41 | 241561.62 |
| Choline | 37002364 | 41496180 | 44547644 | 21541850 | 26234498 | 28474298 |
| Acetylcholine | 10183509 | 8022401.5 | 7439034 | 6465774.5 | 5195340 | 5492373.5 |
| L-Carnitine | 9944727 | 5834613.5 | 6498660 | 4026374.25 | 5016576 | 2895777 |
| AC(2:0) | 27613422 | 44492516 | 55733964 | 18402032 | 19258650 | 29950582 |
| AC(3:0) | 3559881.25 | 5729130 | 5318420 | 4006248 | 4071500.75 | 2726504.75 |
| AC(4:0) | 5946036 | 15163077 | 10879520 | 4671788 | 9153717 | 6749284.5 |
| AC(4-OH) | 443629.03 | 585855.06 | 405480.03 | 342965.5 | 140046.84 | 270003.03 |
| AC(5:0) | 38093668 | 56660576 | 37893916 | 26441722 | 27261608 | 23193990 |
| AC(5:1) | 10974.42 | 18414.76 | 0 | 21237.22 | 0 | 27602.02 |
| AC(5-OH) | 294814.16 | 240683.89 | 257631.69 | 191151.3 | 143680.83 | 67001.12 |
| AC(12:0) | 1208163.38 | 665225.69 | 937726.75 | 405948.75 | 188099.23 | 437924.84 |
| AC(14:0) | 70016552 | 39490640 | 74894176 | 33286158 | 18722904 | 38559180 |
| AC(14:1) | 3064785.75 | 1798710.88 | 1572640.38 | 1139250.75 | 865441.19 | 771982.25 |
| AC(16:0) | 140218000 | 88006344 | 133666312 | 105203144 | 54654816 | 73768192 |
| AC(16:1) | 38269320 | 21214330 | 21013666 | 20760894 | 11413291 | 12939480 |
| AC(18:0) | 17508238 | 9037309 | 20063166 | 12410117 | 5602765.5 | 11188633 |
| AC(18:1) | 56565012 | 24691414 | 39997740 | 43472052 | 15053915 | 19828706 |
| AC(18:2) | 5194334.5 | 2800331 | 2728461.5 | 1614133 | 1227889.5 | 1035939.94 |
| AC(20:4) | 704411 | 316871.81 | 394828.84 | 594556.31 | 254593.88 | 327794.84 |
| FA(5:0) | 156236.42 | 144312.92 | 127202.21 | 132840.02 | 103862.46 | 115946.62 |
| FA(6:0) | 2875723 | 2873619.75 | 2933880 | 3139219.25 | 2173948.75 | 2965069.25 |
| FA(7:0) | 6470022.5 | 6287165.5 | 5888978 | 6068727.5 | 5744289.5 | 5499337.5 |
| FA(8:0) | 26212754 | 19546678 | 22027164 | 23484874 | 25718686 | 22640950 |
| FA(9:0) | 37088340 | 32705786 | 35853180 | 37409704 | 37809932 | 34301196 |
| FA(10:0) | 50669604 | 49397540 | 51346948 | 51439236 | 45746484 | 51323116 |
| FA(12:0) | 49988960 | 47534176 | 53128448 | 53646860 | 46790368 | 54752112 |
| FA(14:0) | 190423344 | 81247496 | 289787872 | 199924096 | 88132472 | 316106208 |
| FA(16:0) | 222733632 | 224826368 | 229958608 | 201662224 | 204596976 | 220977232 |
| FA(18:0) | 127442616 | 150736880 | 131466176 | 109436840 | 102284456 | 108701656 |
| FA(14:1) | 6466426.5 | 3403399.25 | 4102184.25 | 4241406.5 | 2128033.75 | 3694719.25 |
| FA(16:1) | 22216638 | 11194995 | 11878679 | 12686264 | 7571526.5 | 10686475 |
| FA(18:1) | 50579740 | 44427388 | 37248212 | 44658240 | 26941640 | 38994808 |
| FA(18:2) | 6119904 | 4129467.75 | 4260568 | 2772568.75 | 2135865 | 2163577.75 |
| FA(20:4) | 9391800 | 6948138 | 4530362.5 | 6857274 | 3191524.25 | 4082362 |
| FA(20:5) | 284904.78 | 181525.55 | 197473.19 | 284567.78 | 170090.84 | 196023.3 |
| FA(22:6) | 3765173.75 | 3683713.25 | 1912376.62 | 3611305.75 | 1760660.88 | 1465528 |
| FA(20:3) | 2036476.62 | 1608053.88 | 1229679.5 | 1503959.38 | 764281.31 | 724544.5 |
| FA(22:5) | 2317383.75 | 1926749.12 | 1308696.5 | 1882262.5 | 1171359.12 | 1134837.12 |
| Sphingosine | 113321000 | 92899560 | 77503448 | 117547496 | 97843752 | 97417432 |
| Sphinganine 1-phosphate | 237412.62 | 295453.19 | 352472.09 | 294935.5 | 280846.03 | 321216.12 |
| 3-Phospho-D-glyceroyl phosphate | 426856.38 | 197669.7 | 545541.19 | 1085011.12 | 851432.31 | 718968 |

OVCAR3 Supernatant

|  | OVCAR3 Supernatant | | | | | |
| --- | --- | --- | --- | --- | --- | --- |
|  | WT | WT | WT | **KD** | **KD** | **KD** |
| **compound** | **DS2-026-007+** | **DS2-026-008+** | **DS2-026-009+** | **DS2-026-010+** | **DS2-026-011+** | **DS2-026-012+** |
| L-alanine | 9740189 | 4835831.5 | 8792849 | 5281948.5 | 4128233.25 | 6803054 |
| L-arginine | 53894772 | 59394220 | 55913284 | 66667316 | 57683716 | 54659124 |
| L-asparagine | 4029054 | 2660237.75 | 3373054.75 | 2963241.75 | 3049063 | 3235571 |
| L-aspartate | 7318280.5 | 8912445 | 9356801 | 8446141 | 8569705 | 9059345 |
| L-cysteine | 119673.79 | 99164.66 | 198762.86 | 65925.69 | 85201.71 | 142746.08 |
| L-glutamate | 21250060 | 18896776 | 24183544 | 18901590 | 15735365 | 20842234 |
| L-glutamine | 27735134 | 33941100 | 11371651 | 38121852 | 44763956 | 23971538 |
| glycine | 1276194.12 | 858845.44 | 1009045.81 | 862701.56 | 961388.5 | 829133.56 |
| L-histidine | 2106664.75 | 1856498.12 | 2002049.88 | 1951881.12 | 2240773.25 | 2003167.38 |
| L-leucine/isoluecine | 77167392 | 119449920 | 98639240 | 109203608 | 108634720 | 107644632 |
| L-lysine | 11690004 | 12839080 | 11510063 | 11438131 | 11921341 | 9608101 |
| L-methionine | 13214307 | 16781464 | 14439709 | 16511632 | 17297422 | 14581055 |
| L-phenylalanine | 13156985 | 14142373 | 13045104 | 14394567 | 13052937 | 10962996 |
| L-proline | 58386908 | 65505664 | 55787988 | 60462068 | 60588028 | 54004428 |
| L-serine | 1810479.5 | 1015339.69 | 1170929.88 | 1553331.62 | 1451382 | 1530195.88 |
| L-threonine | 3573061.25 | 3630436 | 3125233.25 | 3905591.25 | 4025065.25 | 3087646 |
| L-tryptophan | 26078992 | 31082858 | 26693626 | 33665276 | 26829146 | 21133006 |
| L-tyrosine | 16614795 | 20737058 | 19229138 | 20420978 | 19935222 | 18378118 |
| L-valine | 50781404 | 60845908 | 63817340 | 56611728 | 62708076 | 57852532 |
| L-cystine | 1857906.12 | 1594922.12 | 1719439.88 | 1707297 | 2046783.12 | 2043852.88 |
| GMP | 190921.77 | 0 | 70557.02 | 27150.52 | 0 | 57382.18 |
| Guanine | 310254.41 | 417128 | 351370.41 | 349444.25 | 370962.97 | 345647.72 |
| CDP | 19575.29 | 8085.44 | 34528.17 | 0 | 1969.87 | 22725.91 |
| CMP | 281379.12 | 0 | 198225.33 | 41343.98 | 0 | 123635.21 |
| dCMP | 86166.4 | 84845.15 | 107737.49 | 98497.28 | 84593.79 | 69184.05 |
| Cytidine | 308622.22 | 333493.66 | 253727.83 | 325235.38 | 320863.03 | 229928.7 |
| Cytosine | 147814.3 | 283303.5 | 240980.55 | 278488.78 | 467952.34 | 339581.91 |
| Thymidine | 195170.95 | 126841.09 | 181667.19 | 38873.99 | 135813.28 | 174297.48 |
| UMP | 76603.39 | 16348.57 | 44829.77 | 15582.01 | 37782.28 | 55848.56 |
| Uracil | 143937 | 236130.67 | 288789.03 | 136473.38 | 155971.03 | 196705.08 |
| IDP | 3196252.75 | 55070.21 | 505206.12 | 2144149.75 | 8009.99 | 155583.98 |
| Inosine | 1277238.62 | 802149.44 | 772332 | 950993.69 | 1618810.38 | 1164886.5 |
| Hypoxanthine | 16941606 | 23590518 | 21719592 | 20912860 | 21072604 | 19625798 |
| Xanthine | 211702.67 | 345477.91 | 341559.16 | 273087.97 | 265531.19 | 267424.78 |
| Urate | 817360.69 | 830564.56 | 61113.09 | 610945.5 | 866494.5 | 70054.53 |
| 5-Hydroxyisourate | 381096.28 | 517311.91 | 202703.2 | 581733.19 | 593999.44 | 378323.22 |
| (S)(+)-Allantoin | 630435.06 | 874852.81 | 754173.06 | 744859.81 | 704101.31 | 752782.19 |
| Allantoate | 1167952.88 | 1547427.12 | 2962505.25 | 1186986.62 | 1681321.88 | 2455062.75 |
| 3',5'-Cyclic IMP | 11300047 | 274628.62 | 1922613.5 | 6875134 | 53035.66 | 522998.5 |
| 5-6-Dihydrothymine | 936034.31 | 1315463.88 | 1210629.62 | 1114698.62 | 1426016.62 | 1407823.5 |
| Pyridoxal | 474062.84 | 660664.75 | 832359 | 366340.53 | 491282.97 | 763722.06 |
| 4-Pyridoxate | 172954.81 | 196487.88 | 208358.27 | 221591.44 | 164714.55 | 197381.36 |
| Nicotinamide | 1562590.62 | 2309070.75 | 1947237.12 | 1949677.38 | 2190510.25 | 2056177.38 |
| UDP-glucose | 29551.83 | 4232.37 | 10876.6 | 0 | 0 | 0 |
| ADP-D-ribose | 541006.19 | 805472.06 | 497573.41 | 782655 | 741413 | 619548.94 |
| Phosphate | 201348656 | 222959088 | 208893808 | 204881840 | 202653808 | 186190144 |
| Diphosphate | 23254202 | 31666978 | 25693398 | 27817984 | 29024288 | 25763184 |
| D-Glucose | 38331596 | 33500122 | 33064136 | 43666936 | 45690944 | 37003632 |
| D-Hexose-phosphate | 186518.14 | 130144.06 | 384009.09 | 143689.39 | 131721.05 | 262083.33 |
| D-Glyceraldehyde 3-phosphate/Glycerone phosphate | 248115.73 | 78470.09 | 247766.92 | 78627.15 | 20942.71 | 224547.06 |
| Pyruvate | 7573227.5 | 9585763 | 10062513 | 7452246 | 8735852 | 9565097 |
| Lactate | 247087664 | 260863184 | 350684832 | 221701680 | 205907120 | 297606944 |
| Maltose/Sucrose | 10374758 | 1996595.5 | 4929524.5 | 6243264 | 1850103.38 | 3214967.25 |
| Mannitol/Sorbitol/Glucitol/Iditol | 15041275 | 14409000 | 19291554 | 16362784 | 17894542 | 18155034 |
| Ribose/Ribulose/Arabinose/Xylose/Xylulose | 1604382.12 | 1494786 | 1533339.12 | 1912995.88 | 1976416.12 | 1725205 |
| D-Rhamnose | 235282 | 304211.5 | 262342.75 | 274064.34 | 234312 | 302648.19 |
| D-Arabitol/Xylitol/Ribitol | 535199.88 | 650080.06 | 746711.69 | 682558.12 | 661963.75 | 728107.69 |
| Citrate | 24411360 | 33606620 | 33966932 | 33263584 | 35039476 | 40679076 |
| 2-Oxoglutarate | 1593617.62 | 1681237.38 | 1883112.38 | 1780384.12 | 1348618 | 1864635.12 |
| 2-Oxoglutaramate | 637881.69 | 446853.38 | 615716.31 | 556149.69 | 467437.59 | 765001.94 |
| Succinate | 11177636 | 16399237 | 12487824 | 16781022 | 17888034 | 14672191 |
| Fumarate | 786860 | 605499.81 | 1033269.81 | 891546.94 | 500064 | 733437.75 |
| Malate | 13713969 | 10294849 | 15036899 | 12921704 | 7774604 | 14280400 |
| Oxaloacetate | 44293.72 | 29767.21 | 38933.12 | 77217.66 | 30166.37 | 57351.69 |
| 2-Hydroxyglutarate | 950948.75 | 1265516.38 | 1280884 | 1096324 | 863918 | 1230361.62 |
| 6-Phospho-D-gluconate | 14212.81 | 67571.34 | 46058.15 | 30969.47 | 95544.84 | 74419.4 |
| Glutathione | 29272.08 | 0 | 80917.3 | 0 | 0 | 20646.39 |
| 5-Oxoproline | 28386906 | 50733376 | 45868364 | 37704172 | 46657812 | 49289140 |
| S-Glutathionyl-L-cysteine | 535484.19 | 248921.25 | 566589.31 | 248653.06 | 173365.08 | 319499.41 |
| Ascorbate | 621415.56 | 624031.44 | 288671.28 | 489009 | 651898.38 | 331277.72 |
| Dehydroascorbate | 993610.31 | 971292 | 1130696.88 | 1082250.38 | 970445 | 1220466.62 |
| gamma-L-Glutamyl-D-alanine | 109332.47 | 181519.52 | 147072.62 | 84477.06 | 122331.68 | 133621.64 |
| (5-L-Glutamyl)-L-glutamine | 482478.59 | 681323.75 | 546710.19 | 592762.44 | 667127.31 | 671977.56 |
| Dimethylglycine | 329335.38 | 287156.03 | 301469.16 | 196868.58 | 208210.17 | 309200.31 |
| Folate | 727892.56 | 1112343 | 864877.81 | 958508.19 | 1052737.12 | 848818.75 |
| 10-Formyldihydrofolate | 836852.69 | 84211.61 | 317484.78 | 347505.38 | 133616.39 | 398634.38 |
| 10-Formyltetrahydrofolate | 2875362.25 | 562281.56 | 1622208.5 | 1565866.5 | 413609.16 | 1084094.38 |
| Ornithine | 2092013 | 3428262.75 | 5903364.5 | 2197344.25 | 3618181.75 | 5361436 |
| L-Citrulline | 741991.69 | 772116.69 | 701606.44 | 808405.31 | 855583.44 | 796812.5 |
| Argininosuccinate | 41230.7 | 41076.5 | 62185.1 | 64006.86 | 35056.64 | 34561.71 |
| Spermine | 79558.04 | 106205.79 | 32908.84 | 70853.98 | 39338.44 | 85937.79 |
| N-Acetylneuraminate | 183385.12 | 243045.86 | 202905.58 | 180934.92 | 231536.64 | 220538.8 |
| N-Glycoloyl-neuraminate | 517374.88 | 783436 | 696171.81 | 656762.06 | 657128.12 | 674789.56 |
| UDP-N-acetyl-D-glucosamine | 58066.6 | 4900.81 | 27620 | 19428.6 | 0 | 20806.39 |
| Carnosine | 234204.3 | 329964.16 | 258583.88 | 283370.78 | 283742.97 | 195421.88 |
| Creatine | 28189986 | 30541856 | 24320166 | 31943350 | 33462154 | 27284552 |
| Creatinine | 27657854 | 32225938 | 28953408 | 26844214 | 30880360 | 29921974 |
| 4-Acetamidobutanoate | 165975.58 | 242293.69 | 264837.97 | 183585.31 | 271241.53 | 315957.72 |
| N-Acetyl-L-ornithine | 698784.31 | 630934.06 | 728761.94 | 687332.56 | 518836.59 | 525264.31 |
| trans-4-Hydroxy-L-proline | 15069773 | 15219371 | 14107925 | 12572891 | 16420536 | 16013821 |
| N-Succinyl-L-glutamate 5-semialdehyde | 243942.2 | 634754.75 | 636651.44 | 618177.94 | 324858.28 | 462292.5 |
| Pantothenate | 1034825.5 | 1626370.5 | 1175823.12 | 1340106.62 | 1423855.88 | 1056833.62 |
| Pantetheine | 1220765.38 | 226902.69 | 524509.25 | 1269310.12 | 133527.55 | 341754 |
| Taurine | 273370.44 | 189031.67 | 218093.94 | 258510.5 | 268215.22 | 294420.81 |
| L-Methionine S-oxide | 704180.06 | 419756.59 | 800197.94 | 631771.56 | 496390.5 | 907953.69 |
| Indole-3-acetate | 17574140 | 18322902 | 12035323 | 22500760 | 13603827 | 10609199 |
| Indolepyruvate | 278194.97 | 273789.41 | 170709.3 | 110543.8 | 278077.28 | 296456.03 |
| Kynurenine | 46248.06 | 63109.31 | 71182.56 | 55214.64 | 50817.25 | 57276.53 |
| Kynurenic acid | 188649.02 | 255331.14 | 245648.95 | 175998.38 | 292171.28 | 238777.05 |
| Quinolinic acid | 193434.52 | 18433.54 | 31351.63 | 141852.83 | 101693.62 | 0 |
| Anthranilate | 141492.06 | 637642.12 | 96479.97 | 1191131.38 | 1796891.12 | 528021.94 |
| Picolinic acid | 249711.86 | 292526.75 | 235589.3 | 223985.48 | 335089.53 | 274059.5 |
| g-Oxalo-crotonate | 173586.8 | 739131.31 | 517083.84 | 448608.22 | 194089.55 | 364947.91 |
| 2-Aminomuconate | 473648 | 542963.12 | 734208.56 | 503207.62 | 480238.66 | 492199.59 |
| Hydroxyindole-acetylglycine | 511867.5 | 101552.9 | 108091.45 | 348449.97 | 39248.41 | 72015.07 |
| Ectoine | 225977.75 | 58250.97 | 111393.29 | 169512.19 | 39972.9 | 62136.61 |
| Glycerol 3-phosphate | 211103.94 | 229204.06 | 352454.12 | 256302.5 | 216195.55 | 283978.97 |
| CDP-choline | 183660.44 | 242239.33 | 146496.39 | 367140.16 | 281297.22 | 256457.38 |
| Choline | 7029450 | 31220714 | 10232311 | 14199868 | 19161850 | 5878945.5 |
| Acetylcholine | 1157359.5 | 1289066.62 | 1377960.38 | 1129540.12 | 1498647.5 | 1384446.38 |
| L-Carnitine | 4050535.25 | 5521670.5 | 5509421.5 | 4771530 | 5134027 | 5367392.5 |
| AC(2:0) | 6256502.5 | 9779131 | 8550744 | 6825386.5 | 7922856 | 7087392 |
| AC(3:0) | 2140690.75 | 3599450.75 | 2979467 | 3013711.25 | 3141741.25 | 2633240.25 |
| AC(4:0) | 293645.94 | 485874 | 389373.41 | 382305.59 | 522468.53 | 446950.34 |
| AC(4-OH) | 158518.44 | 160356.48 | 100449.95 | 148724.86 | 142127.91 | 144314.56 |
| AC(4-DC) | 139448.61 | 299778.34 | 201174.14 | 173666.12 | 269996.88 | 234795.17 |
| AC(5:0) | 1587342.38 | 2216739.75 | 2251394.25 | 1580033.38 | 1798333.12 | 1817621.38 |
| AC(6:0) | 16226.36 | 99981.12 | 57710.46 | 44839.56 | 84059.7 | 78179.15 |
| AC(8:0) | 79792.84 | 82321.38 | 67968.2 | 29630.09 | 71585.01 | 51274.78 |
| AC(10:0) | 52996.47 | 94132.16 | 53688.25 | 33577.16 | 59623.41 | 21163.05 |
| AC(10:1) | 220692.61 | 224440.69 | 234686.56 | 257011.08 | 183171.23 | 268453.88 |
| AC(14:0) | 105345.03 | 174540.52 | 185618.33 | 72411.16 | 138143.73 | 55335.58 |
| AC(16:0) | 170408.39 | 384231.5 | 271183.75 | 172610.08 | 357378.41 | 175167.92 |
| AC(16:1) | 95417.62 | 230405.89 | 232481.55 | 193465.7 | 129385.59 | 139654.11 |
| AC(18:0) | 0 | 23434.48 | 21489.43 | 82041.51 | 62079.67 | 14534 |
| AC(18:1) | 48317.06 | 169380.83 | 136037.42 | 147007.77 | 144425.27 | 111631.44 |
| FA(4:0) | 380805.38 | 417157.62 | 334924.91 | 346471.84 | 259013.55 | 324550.28 |
| FA(5:0) | 493894.53 | 649758.81 | 665677 | 300699.41 | 422309.22 | 453201.22 |
| FA(6:0) | 3999782.25 | 4453794.5 | 4213248.5 | 3780179 | 4218276 | 3860444.75 |
| FA(7:0) | 7195897.5 | 6241795.5 | 6992249.5 | 6975548 | 6100834 | 6815020.5 |
| FA(8:0) | 28434342 | 30985094 | 32201538 | 30810638 | 31084934 | 31822054 |
| FA(9:0) | 40968036 | 39957104 | 48418580 | 42740404 | 44025172 | 46209828 |
| FA(10:0) | 67753112 | 65091696 | 59187580 | 62033952 | 64032468 | 63944096 |
| FA(12:0) | 61333332 | 58763252 | 63680780 | 59935996 | 62401588 | 61687328 |
| FA(14:0) | 114239640 | 107114408 | 131357336 | 104802696 | 97492136 | 128248856 |
| FA(16:0) | 241232368 | 287002784 | 227326400 | 243286448 | 232521776 | 190877696 |
| FA(18:0) | 98676064 | 180917072 | 90940248 | 106191192 | 75984256 | 50882756 |
| FA(14:1) | 2184638.25 | 2379807.25 | 1848711.62 | 1515296.5 | 2124038 | 1411408.88 |
| FA(16:1) | 3762304 | 3647934 | 3660478 | 3212660.75 | 3471664.25 | 3095933.75 |
| FA(18:1) | 11077822 | 18687136 | 10133898 | 11828284 | 10002080 | 7868709.5 |
| FA(18:2) | 1376031.12 | 2429270.5 | 1292588.5 | 1489337.88 | 1204673.25 | 930046.44 |
| FA(18:3) | 165001.64 | 207335.7 | 130197.98 | 28335.29 | 126081.77 | 23557.91 |
| FA(20:4) | 499965.75 | 589509.25 | 479134.22 | 497560.88 | 533535.44 | 328028.75 |
| FA(20:5) | 258893.17 | 242969.3 | 235397.92 | 182899.69 | 249460.39 | 290061.47 |
| FA(22:6) | 707612.5 | 1366465.75 | 671724.88 | 1245956.88 | 1009982.94 | 644882.75 |
| FA(20:3) | 86534.13 | 140424.19 | 100108.29 | 80741.62 | 91711.34 | 62554.47 |
| Sphingosine | 137092.27 | 147883.28 | 145263.14 | 141541.83 | 108791.78 | 185341.17 |
