# Supplementary material for: Claudin-4 Modulates Autophagy via SLC1A5/LAT1 as a Mechanism to Regulate Micronuclei
Source: Cancer Res Commun. 2024 Jul 2;4(7):1625–42. doi: 10.1158/2767-9764.CRC-24-0240 (PMC11218812; doi:10.1158/2767-9764.CRC-24-0240)
Supplement: Supplementary Figure 2 — Gating strategy for autophagic flux [file crc-24-0240_supplementary_figure_2_suppsf2.docx]

**Supplementary Figure 2, Villagomez, 2024**


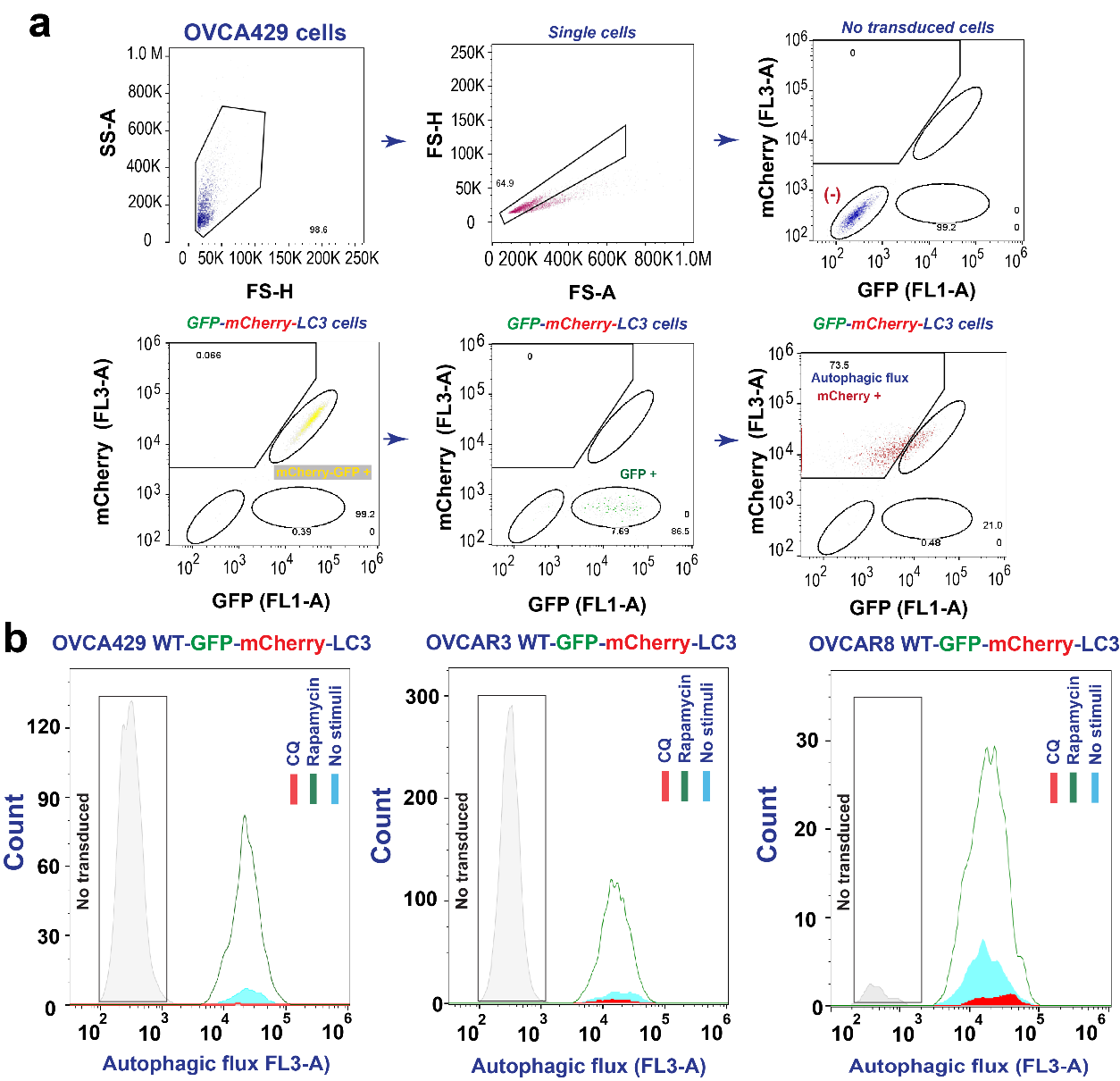


**Supplementary Figure 2. (a)** Autophagy flux strategy for flow cytometry using HGSC cells expressing GFP-mCherry-LC3. **(b)** Confirmation of autophagy flux in HGSC cells- GFP-mCherry-LC3 (OVCAR8 WT, OVCA429 WT, OVCAR3 WT). Chloroquine (CQ) at 40µM for 24h was employed to demonstrate the blocking of autophagy, while rapamycin (rap) at 8µM for 24h was used to illustrate the activation of autophagy.
