# Supplementary material for: Claudin-4 Modulates Autophagy via SLC1A5/LAT1 as a Mechanism to Regulate Micronuclei
Source: Cancer Res Commun. 2024 Jul 2;4(7):1625–42. doi: 10.1158/2767-9764.CRC-24-0240 (PMC11218812; doi:10.1158/2767-9764.CRC-24-0240)
Supplement: Supplementary Table 3 — Survival statistics of claudin-4 with its interacting partners. [file crc-24-0240_supplementary_table_3_suppst3.docx]

**Supplementary Table 3.**

**With Claudin-4**

| Stage of disease | 4 | | 3+4 | | 3 | | | 2+3+4 | | | 2+3 | | |
| --- | --- | --- | --- | --- | --- | --- | --- | --- | --- | --- | --- | --- | --- |
| Groups | **FDR (%)** | **p-value** | **FDR**  **(%)** | **p-value** | **FDR**  **(%)** | **p-value** | | **FDR**  **(%)** | | **p-value** | **FDR**  **(%)** | **p-value** | |
| Cluster 1 | 1 | 8.7e-6 | 1 | 0.0002 | 10 | | 0.0027 | 1 | 7.2e-5 | | 5 | | 0.0011 |
| Cluster 2 | 10 | 0.0022 | 1 | 6.7e-7 | 1 | | 1.2e-6 | 1 | 5.7e-8 | | 1 | | 5.7e-7 |
| Cluster 3 | 50 | 0.0347 | >50 | 0.0458 | >50 | | 0.0402 | >50 | 0.0338 | | 100 | | 0.0921 |
| Cluster 4 | 100 | 0.1271 | 1 | 1.5e-7 | 1 | | 2.3e-7 | 1 | 8.5e-7 | | 1 | | 1.9e-6 |
| All | 5 | 0.0047 | 1 | 0.0002 | 5 | | 0.0009 | 1 | 9.9e-5 | | 5 | | 0.0013 |
