# Supplementary material for: Claudin-4 Modulates Autophagy via SLC1A5/LAT1 as a Mechanism to Regulate Micronuclei
Source: Cancer Res Commun. 2024 Jul 2;4(7):1625–42. doi: 10.1158/2767-9764.CRC-24-0240 (PMC11218812; doi:10.1158/2767-9764.CRC-24-0240)
Supplement: Supplementary Figure 3 — Metabolic enrichments [file crc-24-0240_supplementary_figure_3_suppsf3.docx]

**Supplementary Figure 3, Villagomez, 2024**


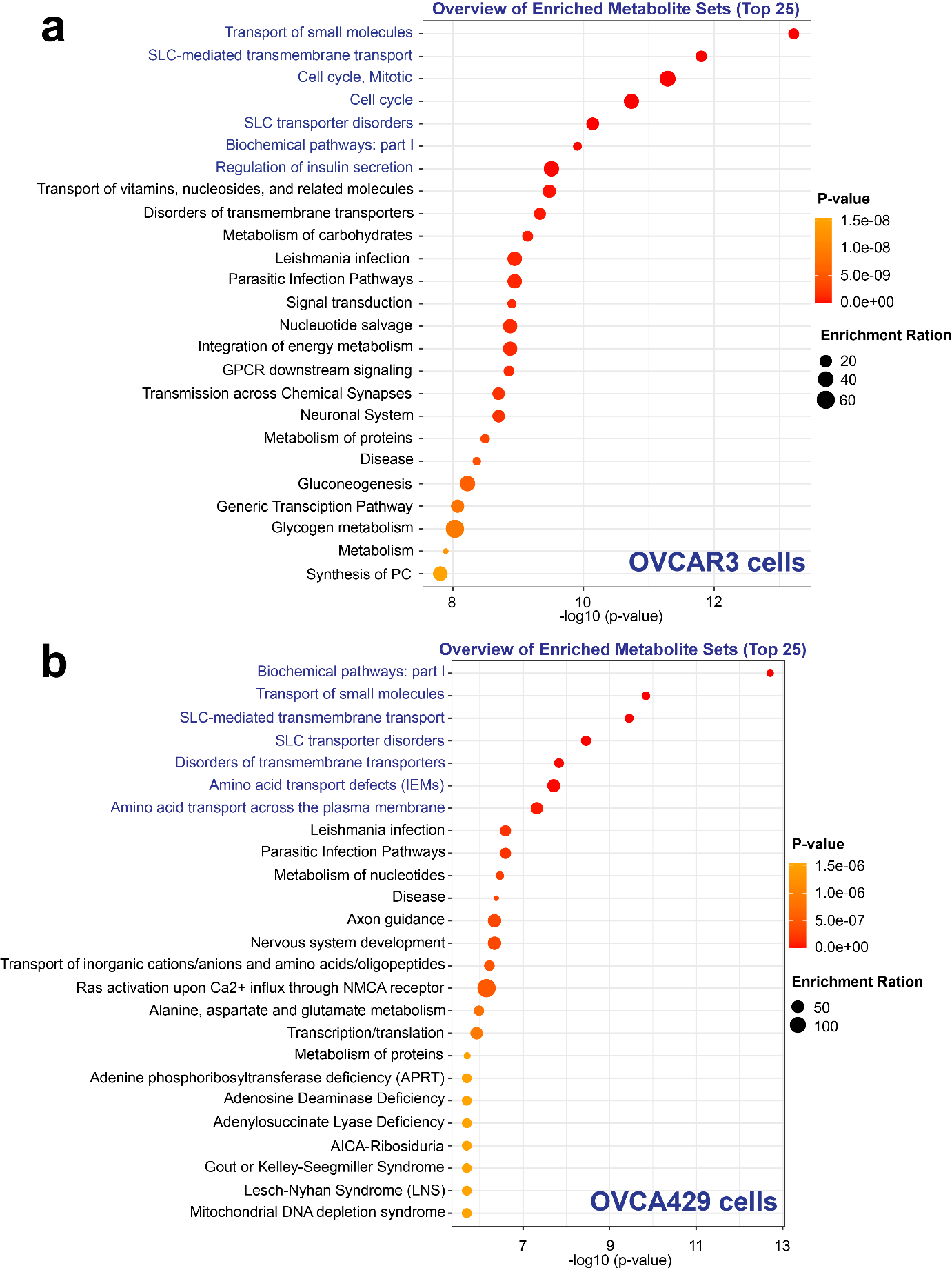
**Supplementary Figure 3 (a)** Enrichment analysis for significant different metabolites (global metabolomics) from claudin-4 downregulation in OVCAR3 and **(b)** OVCA429 (3 independent experiments; significance p<0.05) generated in MetaboAnalyst6.0.
