# Supplementary material for: Claudin-4 Modulates Autophagy via SLC1A5/LAT1 as a Mechanism to Regulate Micronuclei
Source: Cancer Res Commun. 2024 Jul 2;4(7):1625–42. doi: 10.1158/2767-9764.CRC-24-0240 (PMC11218812; doi:10.1158/2767-9764.CRC-24-0240)
Supplement: Supplementary Table 4 — Survival statistics of claudin-4-interacting partners. [file crc-24-0240_supplementary_table_4_suppst4.docx]

**Supplementary Table 4**

**Without Claudin-4**

| Stage of disease | 4 | | 3+4 | | 3 | | | 2+3+4 | | | 2+3 | | |
| --- | --- | --- | --- | --- | --- | --- | --- | --- | --- | --- | --- | --- | --- |
| Groups | **FDR (%)** | **p-value** | **FDR**  **(%)** | **p-value** | **FDR**  **(%)** | **p-value** | | **FDR**  **(%)** | | **p-value** | **FDR**  **(%)** | **p-value** | |
| Cluster 1 | 1 | 8.7e-6 | 1 | 0.0002 | 20 | | 0.0049 | 1 | 6.6e-5 | | 10 | | 0.0019 |
| Cluster 2 | 5 | 0.0019 | 1 | 5.2e-5 | 20 | | 0.0011 | 1 | 3.2e-6 | | 2 | | 8.5e-5 |
| Cluster 3 | 100 | 0.1298 | 100 | 0.0989 | 100 | | 0.1156 | 100 | 0.1689 | | 100 | | 0.2025 |
| Cluster 4 | 100 | 0.1456 | 1 | 1.5e-5 | 1 | | 3.4e-5 | 3 | 0.0002 | | 10 | | 0.0004 |
| All | 20 | 0.0239 | 10 | 0.0016 | 5 | | 0.001 | 10 | 0.002 | | 10 | | 0.0017 |
