# Supplementary material for: Claudin-4 Modulates Autophagy via SLC1A5/LAT1 as a Mechanism to Regulate Micronuclei
Source: Cancer Res Commun. 2024 Jul 2;4(7):1625–42. doi: 10.1158/2767-9764.CRC-24-0240 (PMC11218812; doi:10.1158/2767-9764.CRC-24-0240)
Supplement: Supplementary Figure 4 — Survival analysis in breast, lung, and stomach cancers. [file crc-24-0240_supplementary_figure_4_suppsf4.docx]

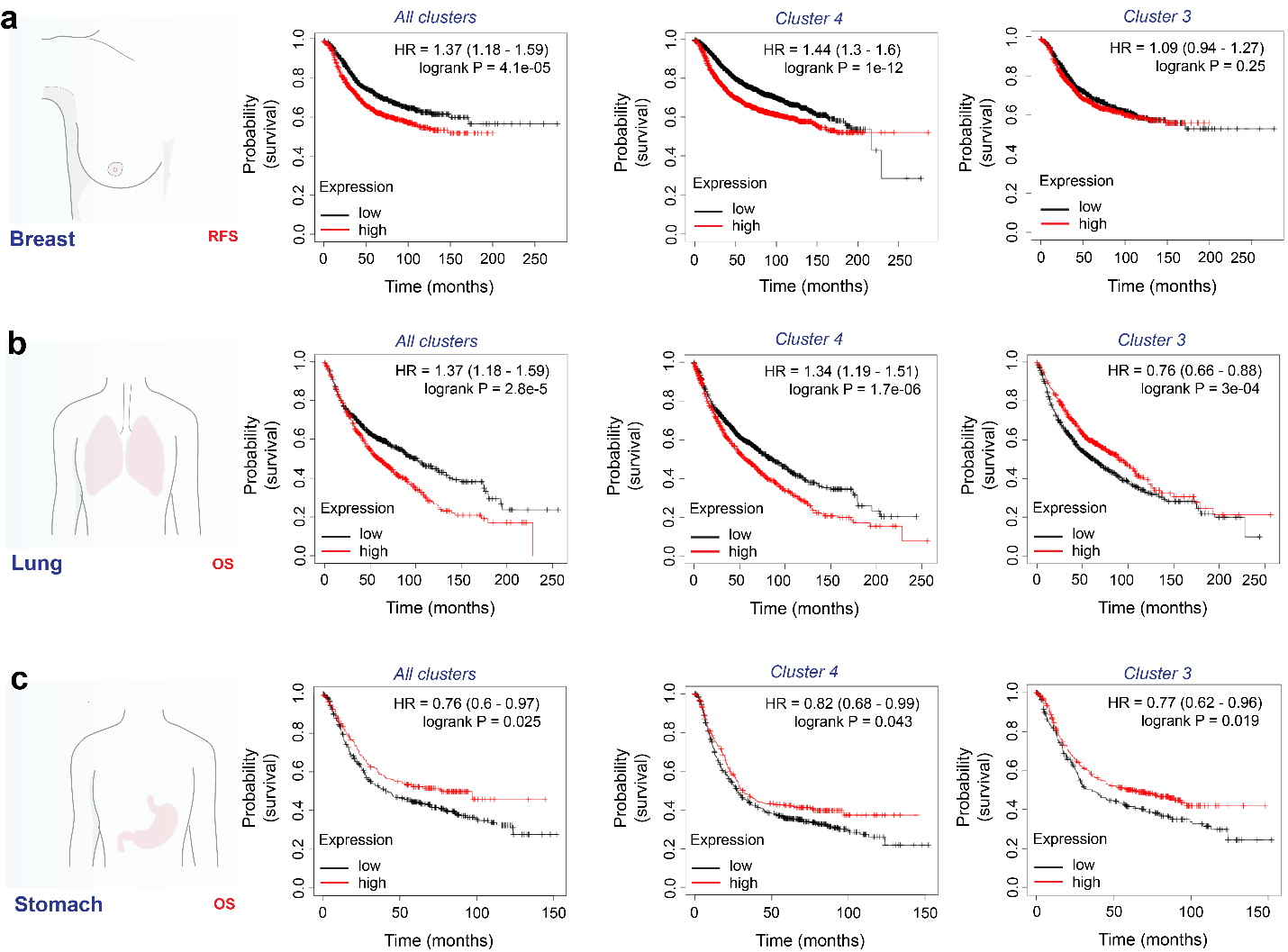
**Supplementary Figure 4, Villagomez, 2024**

**Supplementary Figure 4.** (**a**), correlation analysis of relapse-free survival (RFS) in breast cancer, (**b**) overall survival (OS) in lung, and OS in (**c**) stomach cancer with identified claudin-4 clusters (all, cluster 4 and cluster 3) (significance p<0.05) (Kaplan-Meir Plotter).

**Supplementary bibliographic references:**

1. Gehne, N., Lamik, A., Lehmann, M., Haseloff, R.F., Andjelkovic, A.V., and Blasig, I.E. (2017). Cross-over endocytosis of claudins is mediated by interactions via their extracellular loops. PloS one *12*, e0182106. 10.1371/journal.pone.0182106.

2. Piontek, J., Winkler, L., Wolburg, H., Muller, S.L., Zuleger, N., Piehl, C., Wiesner, B., Krause, G., and Blasig, I.E. (2008). Formation of tight junction: determinants of homophilic interaction between classic claudins. FASEB J *22*, 146-158. 10.1096/fj.07-8319com.

3. Baumgartner, H.K., Beeman, N., Hodges, R.S., and Neville, M.C. (2011). A D-peptide analog of the second extracellular loop of claudin-3 and -4 leads to mislocalized claudin and cellular apoptosis in mammary epithelial cells. Chem Biol Drug Des *77*, 124-136. 10.1111/j.1747-0285.2010.01061.x.

4. Hicks, D.A., Galimanis, C.E., Webb, P.G., Spillman, M.A., Behbakht, K., Neville, M.C., and Baumgartner, H.K. (2016). Claudin-4 activity in ovarian tumor cell apoptosis resistance and migration. BMC Cancer *16*, 788. 10.1186/s12885-016-2799-7.
